# Elucidation of sterol biosynthesis pathway and its co-regulation with fatty acid biosynthesis in the oleaginous marine protist *Schizochytrium* sp.

**DOI:** 10.3389/fbioe.2023.1188461

**Published:** 2023-04-27

**Authors:** Yali Bi, Pengfei Guo, Liangsen Liu, Lei Chen, Weiwen Zhang

**Affiliations:** ^1^ Laboratory of Synthetic Microbiology, School of Chemical Engineering and Technology, Tianjin University, Tianjin, China; ^2^ Frontier Science Center for Synthetic Biology and Key Laboratory of Systems Bioengineering, Ministry of Education of China, Tianjin, China; ^3^ Collaborative Innovation Center of Chemical Science and Engineering, Tianjin, China; ^4^ Center for Biosafety Research and Strategy, Tianjin University, Tianjin, China

**Keywords:** *Schizochytrium*, sterol biosynthesis pathway, genome, inhibitors, co-regulation, fatty acid, carotenoid

## Abstract

Sterols constitute vital structural and regulatory components of eukaryotic cells. In the oleaginous microorganism *Schizochytrium* sp. S31, the sterol biosynthetic pathway primarily produces cholesterol, stigmasterol, lanosterol, and cycloartenol. However, the sterol biosynthesis pathway and its functional roles in *Schizochytrium* remain unidentified. Through *Schizochytrium* genomic data mining and a chemical biology approach, we first *in silico* elucidated the mevalonate and sterol biosynthesis pathways of *Schizochytrium*. The results showed that owing to the lack of plastids in *Schizochytrium*, it is likely to use the mevalonate pathway as the terpenoid backbone pathway to supply isopentenyl diphosphate for the synthesis of sterols, similar to that in fungi and animals. In addition, our analysis revealed a chimeric organization of the *Schizochytrium* sterol biosynthesis pathway, which possesses features of both algae and animal pathways. Temporal tracking of sterol profiles reveals that sterols play important roles in *Schizochytrium* growth, carotenoid synthesis, and fatty acid synthesis. Furthermore, the dynamics of fatty acid and transcription levels of genes involved in fatty acid upon chemical inhibitor-induced sterol inhibition reveal possible co-regulation of sterol synthesis and fatty acid synthesis, as the inhibition of sterol synthesis could promote the accumulation of fatty acid in *Schizochytrium.* Sterol and carotenoid metabolisms are also found possibly co-regulated, as the inhibition of sterols led to decreased carotenoid synthesis through down-regulating the gene *HMGR* and *crtIBY* in *Schizochytrium*. Together, elucidation of the *Schizochytrium* sterol biosynthesis pathway and its co-regulation with fatty acid synthesis lay the essential foundation for engineering *Schizochytrium* for the sustainable production of lipids and high-value chemicals.

## 1 Introduction

Sterols are isoprenoid-derived terpenoids and are essential for the metabolism of eukaryotes, including for the metabolism of crucial plasma membrane components for maintaining membranes in a microfluid state (Benveniste, 2004; Dufourc, 2008). Furthermore, sterols are building blocks for bioactive secondary metabolites and hormones and play a role in signaling and defense pathways (Galea and Brown, 2009; Santner et al., 2009; Smagghe, 2009; Auger et al., 2011). All eukaryotes produce sterols, with the rare exceptions of a few insects, worms, and plant oomycetes, such as *Phytophthora* spp. (Gaulin et al., 2010). The sterol biosynthesis pathway is an example of evolutionary metabolic conservation owing to its long evolutionary history in eukaryotic life and has been extensively studied in vertebrates, fungi, and land plants. A few of the metabolic enzymes of the current sterol pathway are reportedly present in the Last Eukaryotic Common Ancestor (Desmond and Gribaldo, 2009). Generally, sterol biosynthesis begins with the oxidation of squalene to 2,3-epoxysqualene. The resulting 2,3-epoxysqualene is then cyclized into lanosterol or cycloartenol in fungi and animals or higher plants, respectively. Following a succession of redox reactions and demethylation, lanosterol is converted to ergosterol in fungi or cholesterol in animals, respectively, and cycloartenol is converted to phytosterols in higher plants (Benveniste, 2004). In contrast to the well-understood biochemistry and pathway of sterols in model organisms, our understanding of sterol biochemistry and biosynthesis pathway in other organisms, particularly in several families of unicellular eukaryotes, remains lacking.

Co-regulation of fatty acid accumulation and sterol biosynthesis is essential to keep the balance between membrane biosynthesis and turnover during normal cellular growth (Bennett et al., 1995). In animals, the coordinated regulation of sterol and fatty acid accumulation is regulated by a feedback system mediated by sterol regulatory element binding proteins (SREBPs) (Bengoechea-Alonso and Ericsson, 2007). The SREBPs is a conserved family of transcription factors that modulate a series of enzymes involved in biosynthesis for fatty acid, cholesterol, triacylglycerol, and phospholipid, particularly those key enzymes, such as hydroxy-methyl-glutaryl-CoA reductase, HMGR and fatty acid synthase, FAS (Horton et al., 2002a). A previous study showed that the cholesterol content and TAG content in transgenic mice were increased 6- and 22-fold, respectively, through manipulation of SREBPs (Horton et al., 2002b). The co-regulation of sterol and fatty acid accumulation also existed in unicellular microorganisms, and a typical phenotype is the increase in lipid accumulation when sterol synthesis is impaired (Wentzinger et al., 2002; Fabris et al., 2014; Lu et al., 2014). For example, in the oleaginous microalga *Nannochloropsis oceanica*, the addition of squalene epoxidase inhibitor terbinafine impaired the sterol biosynthesis but significantly improved free fatty acid production (Lu et al., 2014). In addition, transcriptional analysis suggested that 1-deoxy-D-xylulose 5-phosphate synthase might contribute to this feedback regulation by sterols (Lu et al., 2014). When the gene *PtOSC* encoding 2,3-oxidosqualene cyclase involved in the sterol biosynthesis pathway was inhibited by RNA interference*,* the sterol content was decreased and the fatty acid accumulation was significantly increased in *Phaeodactylum tricornutum* (Fabris et al., 2014).

*Schizochytrium* sp., one of the nine genera of thraustochytrids, is a unicellular heterotrophic oleaginous marine microorganism that is well known for its ω-3 fatty acids, including docosahexaenoic acid (DHA) (Fossier Marchan et al., 2018; Chi et al., 2022). The fatty acid content of *Schizochytrium* sp. can reach more than 50% of the dry weight, and the DHA content can reach over 57.64% of the total fatty acids (Chang et al., 2013; Wang et al., 2019). In addition to DHA, *Schizochytrium* produces terpenoids, including carotenoids, squalene, and sterols (Du et al., 2021). To date, sterol profiles have only been determined in a few *Schizochytrium* species, including *Schizochytrium* sp. HX-308 (Ren et al., 2015), *Schizochytrium limacinum* B4D1 (Zhu et al., 2022), *Schizochytrium* sp. S31 (Jiang et al., 2020), *Schizochytrium mangrovei* PQ6 (Hang et al., 2019), and *Schizochytrium* sp. MYA1381 (Li et al., 2019). The major sterols include cholesterol, stigmasterol, ergosterol, lanosterol, and cycloartenol. Despite the efforts to chemically describe the sterol content of numerous *Schizochytrium* species, the biochemistry of the *Schizochytrium* sterol biosynthesis pathway remains unclear (Ren et al., 2015; Du et al., 2019; Jiang et al., 2020). The diversity of the sterol species of *Schizochytrium* also rendered the determination of the pathway in the tripartite subdivision difficult. It worth noting that the co-regulation of sterol and fatty acid biosynthesis might also exist in *Schizochytrium*, as the introduction of ω-3 desaturase in *Schizochytrium* sp. HX-308 improved the ratio of ω-3/ω-6 fatty acids, but decreased the contents of unsaponifiable matters such as squalene and sterols (Ren et al., 2015). In addition, overexpression of the acetyl-CoA acetyltransferase gene in *Schizochytrium* sp. HX-308 improved the terpenoid biosynthesis, yet decreased the total fatty acid (TFA) by 71% compared to the control (Huang et al., 2021). Thus, elucidating the sterol pathway and its co-regulation with fatty acid for *Schizochytrium* is necessary and can greatly help to further regulate the fatty acid synthesis and improve lipid accumulation.

The chemical biology approach, also termed as “chemical engineering”, has been widely used to investigate metabolic pathways and regulation mechanisms in plants and algae (Blackwell and Zhao, 2003; Lu et al., 2014; Zhang et al., 2020). It uses cell-permeable small molecules as the modulating ligands for targeting special proteins involved in the metabolic pathway (Blackwell and Zhao, 2003). For example, two inhibitors (mevinolin and fosmidomycin) were used to investigate the terpenoid backbone pathway in *Chromochloris zofingiensis,* wherein mevinolin and fosmidomycin targeted the mevalonate (MVA) and the methylerythritol 4-phosphate (MEP) pathways, respectively (Zhang et al., 2020). A series of sterol biosynthesis inhibitors were also employed to study the sterol biosynthesis pathway in *N. oceanica* (Lu et al., 2014). In addition, the sterol biosynthesis pathway in the diatom *P. tricornutum* was investigated using several inhibitors, including terbinafine, RO 48–8071, and fluconazole, which were specific inhibitors of conventional squalene epoxidase, 2,3-oxidosqualene cyclase, and cytochrome P450 sterol-14-demethylase, respectively (Fabris et al., 2014). For *Schizochytrium* sp. S31 (hereafter *Schizochytrium*), the chemical biology approach may be even more valuable and highly efficient as no effective knockout genetic system exists in *Schizochytrium*. Recently, the whole-genome sequencing of *Schizochytrium* sp. CCTCC M209059 was performed, and the genomic DNA sequences are accessible through the National Library of Medicine (NCBI) (Ji et al., 2015), which can offer a genomic foundation for further understanding the metabolic and regulatory mechanisms in *Schizochytrium.* The carotenoid synthesis pathway has been identified based on the *Schizochytrium* genome database, and the related gene functions were confirmed using enzymatic analysis *in vitro* (Tang et al., 2022).

Herein, we reported the elucidation of the MVA and sterol biosynthesis pathways of *Schizochytrium* using the genome data of *Schizochytrium* sp. CCTCC M209059 (Ji et al., 2015) to identify a set of genes putatively involved in *Schizochytrium* sterol synthesis. Furthermore, the sterol composition and function in *Schizochytrium* growth and the co-regulation of sterol accumulation and fatty acid synthesis were investigated by using specific sterol inhibitors. Our findings expand the understanding of sterol function in *Schizochytrium* and offer valuable insights for *Schizochytrium* engineering for enhanced lipid production or other high-value chemicals.

## 2 Materials and methods

### 2.1 Chemicals

Squalene (B50732), lanosterol (B50780), cholesterol (B20272), and stigmasterol (B20314) were purchased from Yuanye Biotechnology; cycloartenol (C923402) was purchased from Macklin; and mevinolin (PHR1285), fosmidomycin (F8307), terbinafine (T8826), and ketoconazole (PHR1385) were purchased from Merck.

### 2.2 Treatments with chemical inhibitors

*Schizochytrium* sp. S31 (ATCC 20888) was obtained from the American Type Culture Collection (MD, United States) (Zeng et al., 2021). This strain is stored in 20% (v/v) glycerol at −80 °C.

The strain was transferred into 100 mL shake flasks that held 20 mL of seed culture medium and was incubated at 28 °C 180 rpm for 48 h. After that, seed culture (5.0%, v/v) was inoculated into 100 mL shake flasks that held 20 mL fermentation medium and cultivated at 28 °C for 48 h. The composition of seed and fermentation medium was according to the previous study (Zeng et al., 2021). The seed medium contains 5 g/L glucose, 1 g/L yeast extract powder, 1 g/L peptone, and 20 g/L seawater crystals. The fermentation medium contained 40 g/L glucose, 20 g/L sodium glutamate, 0.8 g/L yeast extract powder, 10 g/L Na_2_SO_4_, 0.8 g/L (NH_4_)_2_SO_4_, 4 g/L KH_2_PO_4_.2H_2_O, 0.2 g/L KCl, 4.11 g/L MgSO_4_.7H_2_O, 0.13 g/L CaCl_2_.2H_2_O, 2 mL/L minimal elements (2.6 g/L MnCl_2_, 2.6 g/L ZnSO_4_, 0.4 g/L CuSO_4_, 0.008 g/L Na_2_MoO_4_, 0.4 g/L NiSO_4_, 0.29 g/L FeSO_4_, 0.033 g/L CoCl_2_, 76 mg/L vitamin B_1_, 120 mg/L vitamin B_12_, 256 mg/L C_18_H_32_CaN_2_O_10_).

Two-day-old *Schizochytrium* sp. S31 cultures grown in fermentation medium (Zeng et al., 2021) at 28 °C and 180 rpm were inoculated into 100 mL shake flasks that held 20 mL fermentation medium and treated in triplicate with mevinolin, fosmidomycin, terbinafine, and ketoconazole for 48 h. Following the treatment, the samples were harvested at 12, 24, and 48 h. All the inhibitors were dissolved in dimethyl sulfoxide to prepare the stock solution. The working concentrations of the four inhibitors are listed in Supplementary Table S1.

### 2.3 Determination of dry cell weight and residual glucose

The dry cell weight (DCW) was determined by freeze-drying and weighing (Zeng et al., 2021). Briefly, 2 ml fermentation broth was centrifuged at 12,000 ×*g* for 5 min, washed once with 0.2 M phosphate-buffered saline, and vacuum freeze-dried for 12 h to determine the DCW. The residual glucose concentration of the fermentation medium was analyzed using Glucose Oxidase Assay Kit (Biosino Biotechnology and Science Co., Ltd., China) which is based on the oxidase method. Briefly, 2 µL of the supernatant of the fermentation medium was collected and mixed with 200 µL of the ready-to-use oxidase reagent mixture on a 96-well plate. Then the 96-well plate was placed into the microplate reader, and the program was set to shake at 37 °C for 10 min. The absorbance values were determined at 490_nm_. The double distilled water and glucose standard were used as the negative control and positive control, respectively.

### 2.4 Lipid extraction and analysis

Lipid extraction and analysis were conducted based on a previously described method (Zeng et al., 2021). Briefly, freeze-dried cells (20 mg) were mixed with a reaction solution containing 2 mL of methanol containing 3% (v/v) sulfuric acid and 2 mL of chloroform. The reaction was performed at 97 °C for 2 h, followed by the addition of 3 mL of distilled water. The mixture was vortexed and centrifugated to extract the chloroform phase containing methyl esterification fatty acid. The methyl esterification fatty acids were analyzed by a GC–MS system-GC 7890 coupled to an MSD 5975 (Agilent Technologies, Inc., Santa Clara, CA) equipped with an HP-5MS capillary column (30 m × 0.25 mm × 0.25 µm film; Restek, Bellefonte, PA, United States) according to the previous method (Zeng et al., 2021). Ultra-high purity helium was used as the carrier gas in a constant flow mode of 1 mL/min. The temperature was kept at 80°C for 2 min, and increased to 250°C at a rate of 10 °C/min. When it reached 250°C, the heating rate was decreased to 5 °C/min until 300°C. The temperature was held at 300°C for 10 min before the analysis was terminated. The fatty acids content was calculated using standard curve methods with nonadecanoic acid as an internal standard.

### 2.5 Squalene and sterol extraction and analysis

Squalene was extracted using a previously described method (Li et al., 2020) with some modifications. Briefly, freeze-dried cells (10 mg) were mixed with 600 µl ethyl acetate and 500 mg zirconia beads and homogenized. After centrifuging the mixture at 12,000 ×*g* for 5 min, the upper phase containing squalene was collected for gas chromatography–mass spectrometry (GC–MS) analysis. Squalene was quantified using GC–MS system-GC 7890 coupled with an MSD 5975 (Agilent Technologies, Inc., Santa Clara, CA) equipped with an HP-5MS capillary column (30 m × 0.25 mm × 0.25 µm film; Restek, Bellefonte, PA, United States). Additionally, high-purity helium was used as the carrier gas with a flow rate of 1 mL/min. The oven temperature was started a 70 °C, retained for 1 min, and then increased by 20 °C/min to 280 °C, which was retained for 5 min. The squalene peak was identified via comparison with the commercially available authentic standard.

The cellular sterol content was analyzed as described by Yue et al. (Yue and Jiang, 2009) and Ren et al. (Ren et al., 2009) with some modifications. Freeze-dried cells (100 mg) were resuspended in 500 µL 10% (w/v) KOH-75% (v/v) ethanol solution and 500 mg zirconia beads and homogenized. The broken cells were subsequently saponified using 4 mL 10% (w/v) KOH-75% (v/v) ethanol solution at 60 °C for 15 min. Subsequently, same volume of methanol and ethyl acetate was added to the tubes and heated at 60 °C for 10 min. Additionally, hexane and saturated sodium chloride solution were added and mixed. Following centrifugation, the upper phase containing sterol was collected and evaporated to dryness using a vacuum concentrator system (ZLS-1, Hunan, China). D-sorbitol was used as the internal standard. The residue was silanized before GC analysis. Briefly, the residue was mixed with 20 µl pyridine and 80 µl derivatization reagent (BSTFA +1% TMCS) (Sigma, 15238) and then incubated at 75 °C for 40 min. GC–MS system-GC 7890 coupled with an MSD 5975 (Agilent Technologies, Inc., Santa Clara, CA) equipped with an HP-5MS UI capillary column (30 m × 0.25 mm × 0.25 μm film; Restek, Bellefonte, PA, United States) was used for sterol detection. The high purity helium was used as the carrier gas with the flow rate of 1 mL/min. The oven temperature was started a 170 °C, retained for 2 min, and then increased by 20 °C/min to 280 °C, which was retained for 15 min. The sterols were determined by comparing its retention time and spectrum with those of the authentic standards.

### 2.6 Carotenoid extraction and analysis

The cellular carotenoid content was determined according to a previously described method with minor modifications (Diao et al., 2020). Briefly, freeze-dried cells (50 mg) were mixed with 500 µL acetone and 300 mg zirconia beads and then homogenized. After centrifuging at 12,000 × *g* for 5 min, the upper phase containing the carotenoid was collected for high-performance liquid chromatography (HPLC) analysis.

HPLC analysis was conducted following the previous method (Diao et al., 2020). Briefly, Agilent 1,260 series binary HPLC system (Agilent Technologies, Waldbronn, Germany) equipped with a Symmetry C18 column (5 μm × 4.6/250 mm) (Waters, Milford, MA, United States) was used. Pigments were eluted at a flow rate of 0.7 mL/min with a 25-min gradient of ethyl acetate (0%–100%) in acetonitrile-water-triethylamine (9:1:0.01, vol/vol/vol) and detected using the Agilent diode array detector at 470 nm. Individual carotenoids were identified by their absorption spectra, and their typical retention times were compared with standard samples of pure carotenoids. β-carotene was identified via comparison to commercially available authentic standards β-carotene (SC8140, Solarbio, China).

### 2.7 Data sources, retrieval, and alignment analyses of the sterol biosynthetic enzymes in *Schizochytrium*

The genome of *Schizochytrium* sp. CCTCC M209059 (NCBI accession number: JTFK00000000) (Ji et al., 2015) was used to retrieve the set of genes putatively encoding the sterol biosynthetic enzymes in this study. The *Arabidopsis*, *Saccharomyces cerevisiae*, and *human* protein sequences were retrieved from the UniProt data resource (https://www.uniprot.org/). The proteins from all the genomes were blasted with the *Schizochytrium* genome (Table 1). The correctness of the selected sequences was verified using SMART (Letunic et al., 2012) and the Conserved Domain Database (http://www.ncbi.nlm.nih.gov/cdd/). If there were several alleles, the best-matching allele was selected as the representative for the subsequent analyses. The final sequences selected are listed in Table 1. The sequences were aligned using ClustalW algorithm (http://www.ncbi.nlm.nih.gov/cdd/).

**TABLE 1:** List of genes putatively involved in the terpenoid backbone and sterol biosynthesis pathway of *Schizochytrium.*

Pathways	Gene ID	Category	EC number	Symbol	*Saccharomyces cerevisiae*			*Arabidopsis thaliana*			*Homo sapiens*		
					Gene ID	Identities %	E-value	Gene ID	Identities %	E-value	Gene ID	Identities %	E-value
Terpenoid backbone biosynthesis	KN805403.1_orf00405	Acetyl-CoA acetyltransferase	2.3.1.9	*ACAT*	YPL028W	46	7.00E-98	AT5G47720	51	1.00E-108	hsa:38	50	1.00E-105
	KN805370.1_orf00422	Hydroxymethylglutaryl-CoA (HMG-CoA) synthase	2.3.3.10	*HMGS*	YML126C	45	1.00E-101	AT4G11820	47	1.00E-110	hsa:3157	48	1.00E-113
	KN805388.1_orf00290	Hydroxymethylglutaryl-CoA (HMG-CoA) reductase	1.1.1.34	*HMGR*	YLR450W	44	1.00E-105	AT1G76490	51	1.00E-110	hsa:3156	49	1.00E-104
	KN805401.1_orf00324	Mevalonate kinase	2.7.1.36	*MK*	YMR208W	24	1.00E-03	AT5G27450	22	4.00E-03	hsa:4598	28	1.00E-05
	KN805371.1_orf01820	Phosphomevalonate kinase	2.7.4.2	*PMK*	YMR220W	32	8.00E-31	AT1G31910	35	7.00E-53	hsa:10654	-	-
	KN805434.1_orf00223	Diphosphomevalonate decarboxylase	4.1.1.33	*MPDC*	YNR043W	45	1.00E-84	AT2G38700	45	1.00E-87	hsa:4597	49	1.00E-93
	KN805371.1_orf02895	Isopentenyl-diphosphate Delta-isomerase	5.3.3.2	*IPI*	YPL117C	46	1.00E-50	AT3G02780	50	8.00E-55	hsa:3422	50	1.00E-58
	KN805450.1_orf00079	Geranyl-diphosphate synthase	2.5.1.1	*GPS*	YPL069C	23	5.00E-09	AT2G34630	40	2.00E-67	has:9453	-	-
	KN805379.1_orf00342	Bifunctional (2E,6E)-farnesyl pyrophosphate synthase/dimethylallyltranstransferas	2.5.1.1/2.5.1.10	*FPS*	YJL167W	46	2.00E-84	AT4G17190	45	2.00E-78	hsa:2224	44	8.00E-78
	not found	1-deoxy-D-xylulose 5-phosphate synthase	2.2.1.7	*DXS*	-	-	-	AT3G21500/AT4G15560/AT5G11380	-	-	-	-	-
	not found	1-deoxy-D-xylulose 5-phosphate reductoisomerase	1.1.1.267	*DXR*	-	-	-	AT5G62790	-	-	-	-	-
	not found	2-C-methyl-D-erythritol 4-phosphate cytidylyltransferase	2.7.7.60	*ISPD*	-	-	-	AT2G02500	-	-	-	-	-
	not found	4-diphosphocytidyl-2-C-methyl-D-erythritol kinase	2.7.1.148	*CDPMEK*	-	-	-	AT2G26930	-	-	-	-	-
	not found	2-C-methyl-D-erythritol 2,4-cyclodiphosphate synthase	4.6.1.12	*ISPF*	-	-	-	AT1G63970	-	-	-	-	-
	not found	4-hydroxy-3-methylbut-2-enyl diphosphate synthase	1.17.7.1/1.17.7.3	*HDS*	-	-	-	AT5G60600	-	-	-	-	-
	not found	4-hydroxy-3-methylbut-2-en-1-yl diphosphate reductase	1.17.7.4	*HDR*	-	-	-	AT4G34350	-	-	-	-	-
Sterol biosynthesis	KN805399.1_orf01057	Squalene synthase	2.5.1.21	*SQS*	YHR190W	37	1.00E-51	AT4G34640	37	3.00E-46	hsa:2222	35	5.00E-54
	not found	Squalene epoxidase	1.14.14.17	*SQE*	YGR175C	-	-	AT1G58440/AT2G22830/AT4G37760/AT5G24140/AT5G24150/AT5G24160	-	-	hsa:6713	-	-
	KN805388.1_orf00276	Lanosterol synthase	5.4.99.7	*LAS*	YHR072W	37	1.00E-120	AT3G45130	42	1.00E-157	hsa:4047	42	1.00E-156
	KN805388.1_orf00276	Cycloartenol synthase	5.4.99.8	*CAS*	-	-	-	AT2G07050	43	1.00E-172	-	-	-
	KN805454.1_orf00029	Sterol 14-demethylase	1.14.14.154/1.14.15.36	*CYP51G1*	YHR007C	30	4.00E-62	AT1G11680	41	1.00E-101	hsa:1,595	35	1.00E-79
	KN805390.1_orf00068	Δ-(14)-Sterol reductase	1.3.1.70	*FK*	YNL280C	40	1.00E-86	AT3G52940	34	4.00E-54	hsa:3930	46	2.00E-98
	KN805429.1_orf00117	Methylsterol monooxygenase	1.14.18.9	*SMO*	YGR060W	28	4.00E-17	-	-	-	hsa:6307	34	3.00E-13
	KN805374.1_orf01215	Sterol-4-α-carboxylate 3-dehydrogenase	1.1.1.170	*NSDHL*	YGL001C	28	6.00E-30	0.00E+00			hsa:50814	33	6.00E-48
	KN805408.1_orf00240	3-Keto-steroid reductase	1.1.1.270	*HSD17B7*	YLR100W	23	2.00E-03	-	-	-	hsa: 51478	18	7.00E-05
	KN805382.1_orf01293	24-Methylenesterol C-methyltransferase	2.1.1.41	*SMT*	YML008C	36	5.00E-55	AT5G13710	39	9.00E-61	-	-	-
	KN805382.1_orf01293	24-Methylenesterol C-methyltransferase	2.1.1.41	*SMT*	YML008C	36	5.00E-55	AT1G20330	34	1.00E-48	-	-	-
	KN805382.1_orf01293	24-Methylenesterol C-methyltransferase	2.1.1.41	*SMT*	YML008C	36	5.00E-55	AT1G76090	33	1.00E-46	-	-	-
	KN805384.1_orf00665	Δ-7-Sterol 5-desaturase	1.14.19.20	*STE1*	YLR056W	45	3.00E-58	AT3G02580	28	7.00E-18	hsa:6309	54	1.00E-89
	KN805393.1_orf00257	C-22 Sterol desaturase	1.14.19.41	*CYP710A*	YMR015C	24	1.00E-26	AT2G28850	30	5.00E-55	-	-	-
	KN805390.1_orf00068	Δ-(24 (241)))-Sterol reductase	1.3.1.71	*ERG4*	YGL012W	30	1.00E-50	-	-	-	-	-	-
	KN805380.1_orf01851	Δ-24-Sterol reductase	1.3.1.72	*DWF1*	-	-	-	AT3G19820	34	2.00E-70	hsa:1718	46	1.00E-107
	KN805386.1_orf01801	cholestenol Δ-isomerase	5.3.3.5	*HYD1*	-	-	-	AT1G20050	20	4.40E-01	hsa:10682	23	6.00E-03
	KN805434.1_orf00456	7-Dehydrocholesterol reductase	1.3.1.21	*DWF5*	-	-	-	AT1G50430	47	1.00E-103	hsa:1717	32	3.00E-62
	KN805429.1_orf00117	4,4-Dimethylsterol C-4α-methyl-monooxygenase	1.14.18.10	*SMO*	-	-	-	AT4G12110	30	2.00E-17	-	-	-
	KN805429.1_orf00117	4α-Monomethylsterol monooxygenase	1.14.18.11	*SMO*	-	-	-	AT1G07420	33	4.00E-24	-	-	-
	KN805375.1_orf02114	Cycloeucalenol cycloisomerase	5.5.1.9	*CPI1*	-	-	-	AT5G50375	30	7.00E-26	-	-	-

### 2.8 Quantitative real time polymerase chain reaction

Quantitative real time polymerase chain reaction (qRT-PCR) was performed according to a previously described method (Wang et al., 2019) with some modifications. Briefly, the cells (5 OD_660_ units) were harvested at different time points and resuspended in Trizol reagent (15596018, Invitrogen, Camarillo, CA, United States). Subsequently, the total RNA was extracted using Direct-zol™ RNA MiniPrep (R2050, Qiagen, Valencia, CA, United States), and the subsequent synthesis of cDNA was achieved using A HiScript^®^ II Q RT SuperMix for qPCR (+gDNA wiper) (R223-01, Vazyme, China). The cDNA was used as the template for qRT-PCR, and the primers are listed in Supplementary Table S2. The relative abundance of the different mRNA molecules was estimated according to a previously described method (Livak and Schmittgen, 2001).

### 2.9 Statistical analyses

Three biological replicates were performed for all the experiments. The results were expressed as mean ± standard deviation (m ± SD). Paired Student’s *t-*tests were used for statistical analyses via SPSS. Differences with a *p*-value of <0.05 or *<*0.01 were considered significant.

## 3 Results

### 3.1 Sterol accumulation pattern during the growth of *Schizochytrium*

**FIGURE 1:**
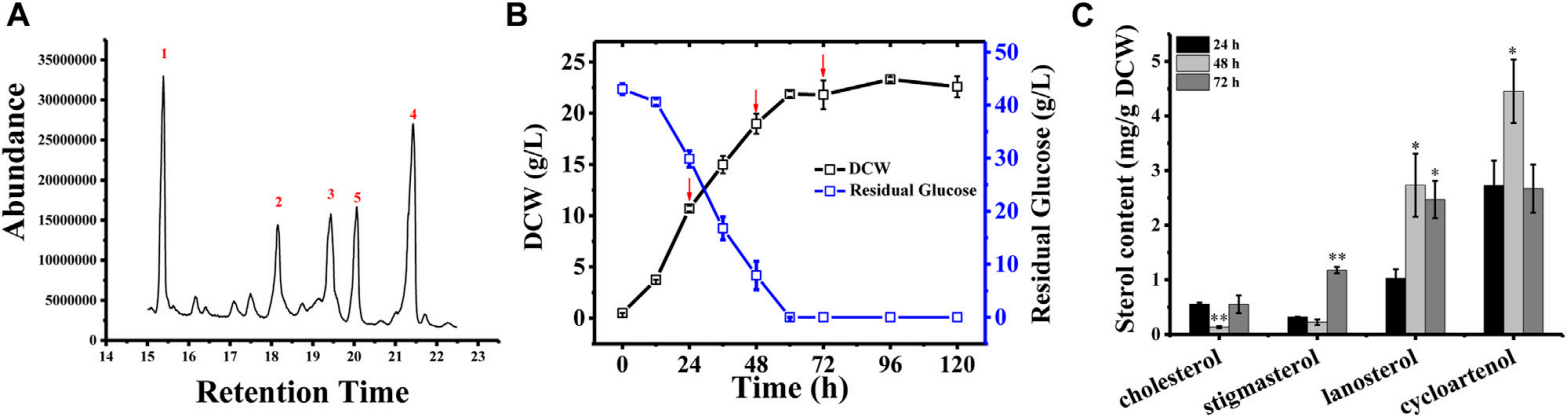
Sterol composition and profile in *Schizochytrium* sp. S31 growth. **(A)**, GC-MS chromatogram of a TMS-derived extract from a 1-day-old *Schizochytrium* culture. **(B)**, the growth curve and residual glucose during the fermentation. **(C)**, changes of the sterols of *Schizochytrium* during growth stages (24, 48, and 72 h). Data represent means ± SD (n = 3). Asterisks (*) indicate *p* values < 0.05. Asterisks (**) indicate *p* values < 0.01.

In *Schizochytrium* sp. S31, four major sterols, including cholesterol, stigmasterol, lanosterol, and cycloartenol, were identified and analyzed using GC–MS (Figure 1A and Supplementary Figure S1). However, ergosterol has been detected in *Schizochytrium* sp. S31 using ultraperformance liquid chromatography method in a previous study (Jiang et al., 2020). We speculated that the failure of ergosterol detection in this study might be due to technical limitations and different culture conditions. To investigate the sterol accumulation patterns, *Schizochytrium* cells were examined for 5 days. From the cell growth curve of *Schizochytrium,* we could see that glucose was exhausted after 60 h and the growth of *Schizochytrium* approached the stationary phase (Figure 1B). Sterols were identified and analyzed in the three time points (*i.e.,* 24, 48, and 72 h) (Figure 1C). Sterol analysis revealed that cholesterol and stigmasterol levels exhibited a moderate decrease at 48 h of *Schizochytrium* cultures and then increased rapidly at 72 h, reaching 0.55 mg/g DCW and 1.17 mg/g DCW, respectively (Figure 1C). Conversely, both of the intermediate metabolites lanosterol and cycloartenol levels rapidly increased at 48 h, consequently decreasing at 72 h, reaching 2.47 mg/g DCW and 2.66 mg/g DCW, respectively (Figure 1C). The dynamic changes of the intermediate metabolites (lanosterol and cycloartenol) might support the presence of *de novo* lanosterol synthesis and cycloartenol synthesis for sustaining continued cholesterol and stigmasterol accumulation, respectively. The results are consistent with the previous study in *Schizochytrium* sp. HX-308, in which the dynamic changes of sterols in the fed-batch fermentation of *Schizochytrium* sp. HX-308 suggested that the cholesterol and stigmasterol were mainly accumulated at the late fermentation stage (168–192 h), and the other sterols, including β-sitosterol, lanosterol, and cycloartenol, were mainly accumulated at the early stage of the fermentation (Ren et al., 2014). These results indicate that the synthesis and accumulation of sterol in *Schizochytrium*, including those of cholesterol and stigmasterol, appear to be the features of late cell growth.

### 3.2 *In silico* construction of the *Schizochytrium* sterol pathway

We mined the genome of *Schizochytrium* sp. CCTCC M209059 (Ji et al., 2015) to identify the set of genes putatively encoding the biosynthetic enzymes involved in terpenoid backbone synthesis and sterol biosynthesis in *Schizochytrium*. The identified genes are listed in Table 1.

**FIGURE 2:**
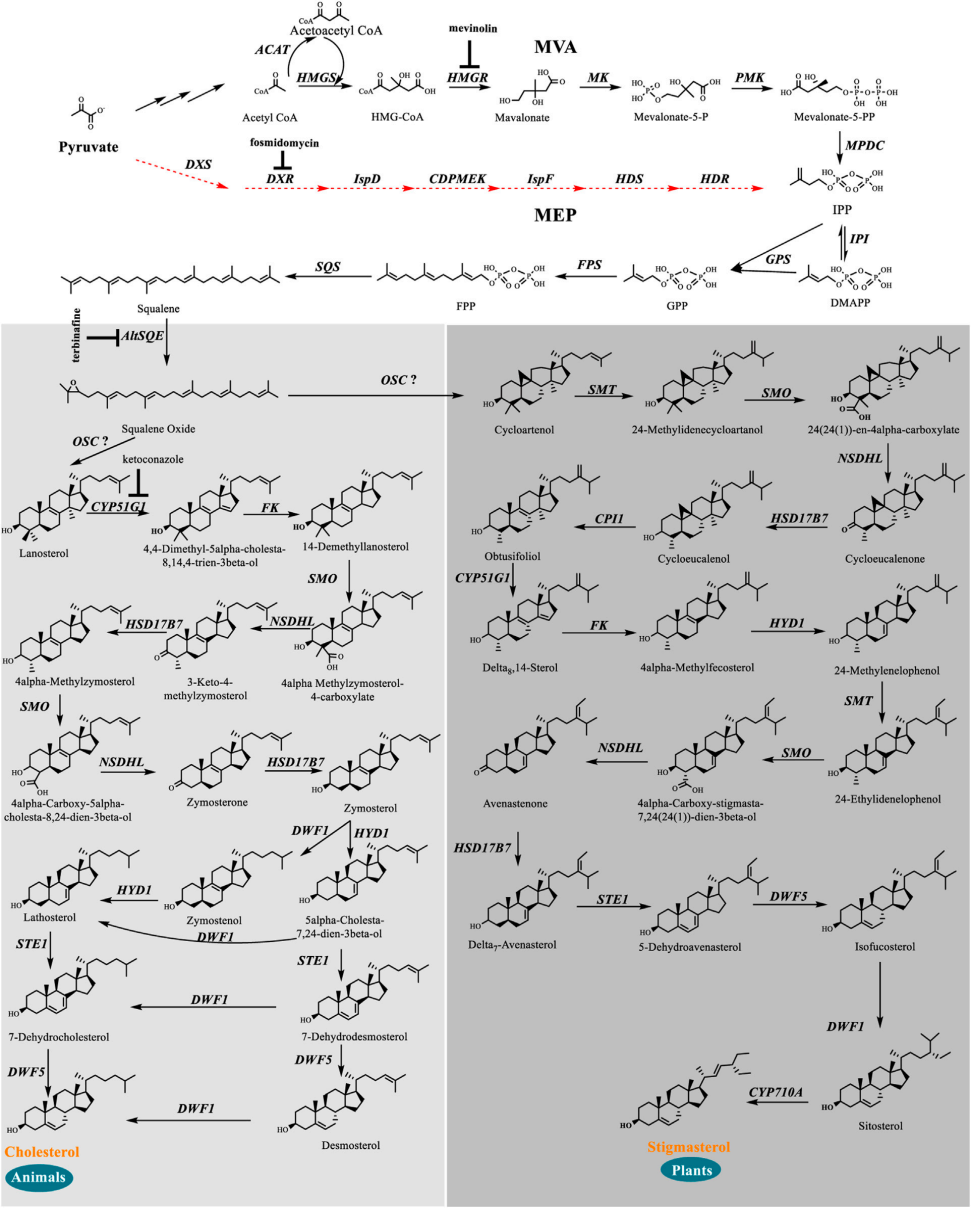
*In silico* construction of the steroid backbone biosynthesis and sterol biosynthesis pathway in *Schizochytrium*. Specific inhibitors are underlined. Black plain arrows indicate genes were identified in *Schizochytrium* genome. Red dashed arrows indicate genes were not identified in *Schizochytrium* genome. The symbol”?” represents uncertainty. Abbreviations: HMG-CoA, hydroxy-methyl-glutaryl-coenzyme A; Mevalonate-5-P, mevalonate-5-phosphate; Mevalonate-5-PP, 5-diphosphomevalonate; IPP, isopentenyl diphosphate; DMAPP, dimethylallyl diphosphate; GPP, geranyl pyrophosphate; FPP, farnesyl pyrophosphate; *HMGS*, HMG-CoA synthase; *HMGR*, HMG-CoA reductase; *MK*, mevalonate kinase; *PMK*, phosphomevalonate kinase; *MPDC*, diphosphomevalonate kinase; *IPI*, isopentenyl diphosphate isomerase; *GPS*, geranyl diphosphate synthase; *FPS*, farnesyl pyrophosphate synthase; *DXS*, 1-deoxy-D-xylulose 5-phosphate synthase; *DXR*, 1-deoxy-D-xylulose 5-phosphate reductoisomerase; *ISPD*, 2-C-methyl-D-erythritol 4-phosphate cytidylyltransferase; *CDPMEK*, 4-diphosphocytidyl-2-C-methyl-D-erythritol kinase; *ISPF*, 2-C-methyl-D-erythritol 2,4-cyclodiphosphate synthase; *HDS*, 4-hydroxy-3-methylbut-2-enyl diphosphate synthase; *HDR*, 4-hydroxy-3-methylbut-2-enyl diphosphate reductase; *SQS*, squalene synthase; *AltSQE*, squalene epoxidase; *OSC*, 2,3-oxidosqualene cyclase; *CYP51G1*, sterol 14-demethylase; *FK*, Δ-(14)-sterol reductase; *NSDHL*, sterol-4-α-carboxylate 3-dehydrogenase; *HSD17B7*, 3-keto-steroid reductase; *SMT*, 24-methylenesterol C-methyltransferase; *STE1*, Δ-7-sterol 5-desaturase; *CYP710A*, C-22 sterol desaturase; *DWF1*, Δ-24-sterol reductase; *HYD1*, cholestenol Δ-isomerase; *DWF5*, 7-dehydrocholesterol reductase; *SMO*, 4α-monomethylsterol monooxygenase; *3BETAHSD*, 3β-hydroxysteroid-4α-carboxylate 3-dehydrogenase; *CPI1*, cycloeucalenol cycloisomerase.

We identified a complete set of genes encoding the enzymes involved in the MVA pathway and sterol biosynthesis in *Schizochytrium* (Table 1; Figure 2). Starting from farnesyl pyrophosphate, 19 genes (Table 1) that encode enzymes required for the formation of sterols, including stigmasterol and cholesterol, were indicated to be involved in the *Schizochytrium* sterol biosynthesis pathway. These enzymes enable various speculative pathway reconstructions, thereby rendering the creation of artificial versions of the natural pathways in plants, fungi, and animals potentially feasible (Table 1; Figure 2). The constructed sterol biosynthesis pathway of *Schizochytrium* demonstrated features of both algae and animals. However, no conserved squalene epoxidase (*SQE*, EC 1.14.14.17) orthologs were identified in the genome of *Schizochytrium*, consistent with the findings of a previous study in the *Hondaea fermentalgiana* strain CCAP 4062/3 (Dellero et al., 2018). Among the identified genes, we identified a single 2,3-oxidosqualene cyclase (OSC) encoded by ORF KN805388.1_orf0027. The overall structure of the steroid biosynthesis pathway in *Schizochytrium* and these observations were further investigated.

The constructed sterol biosynthesis pathway of *Schizochytrium* revealed features of algae. Three sterol methyltransferase (SMT) enzymes are present in the plant *Arabidopsis thaliana* and can convert various substrates into either methylated (SMT1) or ethylated (SMT2 or SMT3) phytosterols (Carland et al., 2010). Conversely, numerous algae have only one copy of the candidate gene encoding SMT that potentially catalyzes the methylation reaction yielding methylated and ethylated phytosterols, for example, in the green algae *Chlamydomonas reinhardtii*, *Chlorella variabilis*, and in the diatom *P. tricornutum* (Lu et al., 2014). In *S. cerevisiae*, only one copy of the *Erg6* gene reportedly encodes the sterol 24-C-methyltransferase, which catalyzes a single methyl addition (McCammon et al., 1984). In the *Schizochytrium* genome, only one candidate gene KN805382.1_orf01293 encoding SMT was identified to catalyze the methylation reactions, yielding methylated and ethylated products that resembled those of algae.

The constructed *Schizochytrium* sterol biosynthesis pathway also exhibited characteristics of those found in animals. The key enzyme catalyzing the reduction of the sterol side chain from *Arabidopsis* and higher plants differed from that of animals and fungi. In *Arabidopsis*, the sterol 24 (28) isomerase-reductase (DWF1) is necessary for both the isomerization and reduction of 24-methylenecholesterol (Choe et al., 1999). However, the equivalent enzymes Δ-(24)-sterol reductase encoded by *DHCR24* in animals and Δ-(24 (24(1)))-sterol reductase encoded by *erg4* in yeast can only catalyze the reduction of the Δ-24 double bond of sterol intermediates during cholesterol and ergosterol biosynthesis (Zweytick et al., 2000; Waterham et al., 2001). According to the analysis of amino acid sequence alignment, *Schizochytrium* Δ-24-sterol reductase is more similar to *Homo sapiens* DHCR24 than to *Arabidopsis* DWF1 (Table 1). The gene encoding 24 (25) reductase in the oleaginous microalgal *N. oceanica* was also more similar to *H. sapiens* DHCR24, consistent with the results of this study (Lu et al., 2014)*.* Therefore, the evidence suggested that the *Schizochytrium* sterol pathway also exhibits characteristics of the pathway in animals.

In addition, we employed a chemical biology approach to explore the structure of the sterol metabolic pathways and its co-regulation with fatty acid accumulation, wherein *Schizochytrium* was treated with a series of sterol biosynthetic inhibitors (Figure 2 and Supplementary Table S1) to simulate the phenotype of the metabolic product biosynthesis. This is a powerful tool for clarifying the biological synthesis and functions of metabolites (Blackwell and Zhao, 2003; Hicks and Raikhel, 2009), particularly as no efficient targeted gene knockout system existed in *Schizochytrium* sp. S31 by now. The chemical inhibitors that we used have been well studied, and specificities to their corresponding enzymes have been established (Alberts et al., 1980; Hagen and Grunewald, 2000; Warrilow et al., 2010; Pollier et al., 2019).

### 3.3 Cytosolic MVA pathway for terpenoid biosynthesis in *Schizochytrium*

In land plants, there are two different pathways for isopentenyl diphosphate (IPP)/dimethylallyl diphosphate (DMAPP) biosynthesis: the cytosolic MVA pathway starting with acetyl-coenzyme A (CoA) and the plastid MEP pathway starting involving pyruvate and glyceraldehyde-3-phosphate (Vranova et al., 2013). The evolutionary history of the enzymes involved in these two pathways and the phylogenetic distribution of their genes in the genome suggests that the MVA and MEP pathways are closely related to archaebacteria and eubacteria, respectively (Lange et al., 2000). The emergence of the MEP pathway–special genes is limited to eukaryotes carrying plastids, which indicates that these genes were obtained from the cyanobacterial ancestor of plastids (Lange et al., 2000). The MEP pathway and not the MVA pathway was considered to contribute to the synthesis of IPP/DMAPP precursors for carotenoid biosynthesis in the green algae *Haematococcus pluvialis* and *C. reinhardtii* (Hagen and Grunewald, 2000; Lohr et al., 2005). Conversely, the MVA pathway rather than the MEP pathway has been suggested to occur in thraustochytrid *Aurantiochytrium limacinum* ATCC MYA-1381 (Dellero et al., 2018) and diatom *P. tricornutum* (Fabris et al., 2014).

**FIGURE 3:**
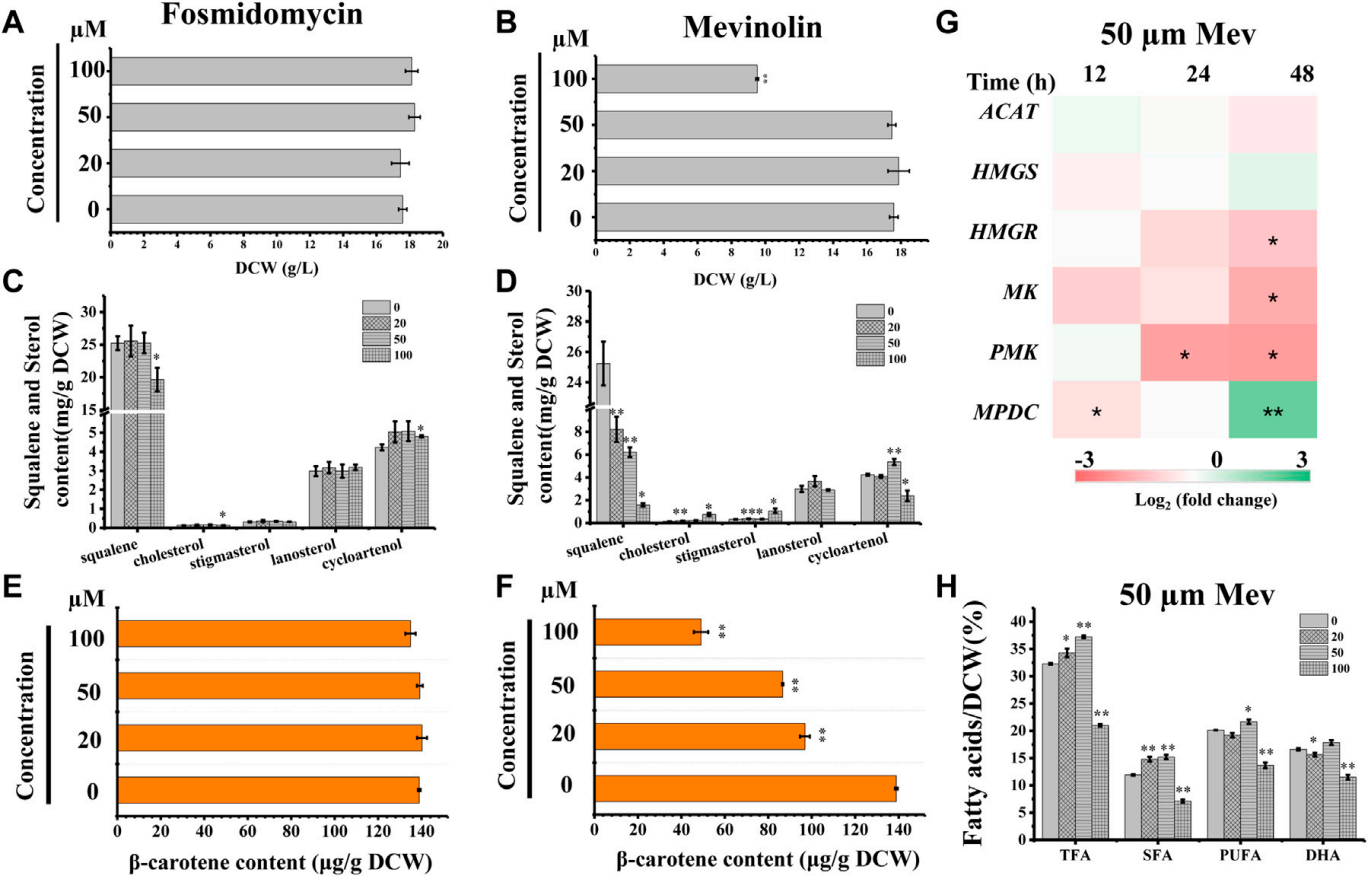
Effects of mevinolin and fosmidomycin on growth, sterols, carotenoid, and lipids in *Schizochytrium* sp. S31. **(A)** and **(B)**, DCW as affected by different concentrations (micromolar) of fosmidomycin **(A)** and mevinolin **(B)**. **(C)** and **(D)**, sterols as affected by different concentrations (micromolar) of fosmidomycin **(C)** and mevinolin**(D)**. **(E)** and **(F)**, β-carotene as affected by different concentrations (micromolar) of fosmidomycin **(E)** and mevinolin**(F)**. **(G)** heatmap showing the transcript dynamics of selected genes involved in MVA pathway under the treatment of 50 µM mevinolin. The data are expressed as log_2_ (fold change) values of transcripts relative to the control at 12 h, 24 h, and 48 h, determined by qRT-PCR. Time refers to the duration of the induction conditions. **(H)** fatty acids as affected by different concentrations (micromolar) of mevinolin. Mev, mevinolin. Data represent means ± SD (n = 3). Asterisks (*) indicate *p* values <0.05. Asterisks (**) indicate *p* values <0.01. *ACAT*, acetyl-CoA acetyltransferase; *HMGS*, hydroxy-methyl-glutaryl-CoA synthase; *HMGR*, hydroxy-methyl-glutaryl-CoA reductase; *MK*, mevalonate kinase; *PMK*, phosphomevalonate kinase; *MPDC*, diphosphomevalonate kinase.

We searched the genome of *Schizochytrium* sp. CCTCC M209059 and identified all the genes involved in the MVA pathway. However, it lacks certain genes involved in the MEP pathway (Figure 2; Table 1), consistent with the results of previous studies involving other thraustochytrids (Dellero et al., 2018; Park et al., 2018; Ye et al., 2019; Zhang et al., 2019; Du et al., 2021). Two inhibitors (mevinolin and fosmidomycin) were applied to *Schizochytrium* cultures during fermentation. Mevinolin and fosmidomycin target *HMGR* in the MVA pathway and 1-deoxy-D-xylulose 5-phosphate reductoisomerase (*DXR*) in the MEP pathway (Alberts et al., 1980; Hagen and Grunewald, 2000). Fosmidomycin demonstrated no significant impact on cell growth and β-carotene and sterols accumulation even when applied at 100 µM (Figures 3A,C,E). Conversely, mevinolin affected the accumulation of both β-carotene and squalene in a concentration-dependent manner, thereby decreasing 64.69% and 93.70% β-carotene and squalene, respectively, at a concentration of 100 µM (Figures 3D,F). This might be due to the strong inhibition of HMGR activity, leading to the decreased supply of the common precursor IPP of sterol biosynthesis and carotenoid biosynthesis pathway. In addition, the sterol profile in *Schizochytrium* substantially altered the following treatment with mevinolin (Figure 3D). Both cholesterol and stigmasterol contents were increased in a concentration-dependent manner following treatment with mevinolin, leading to significant 4.51- and 2.39-fold increases compared to the control, respectively, at a concentration of 100 µM (Figure 3D). Conversely, both of the two intermediates (*i.e.*, lanosterol and cycloartenol) in the cholesterol and stigmasterol pathways were significantly decreased with the addition of 100 µM mevinolin, which might be due to the joint effects of the decreased supply of the precursor squalene and the elevated synthesis of the end products (*i.e.*, cholesterol and stigmasterol), as squalene is generally as the common precursor of the lanosterol and cycloartenol synthesis, and lanosterol and cycloartenol are the precursors of the cholesterol and stigmasterol synthesis pathways (Figure 2) (Benveniste, 2004; Kaur et al., 2011).

To understand the expression mode of the *Schizochytrium* MVA pathway, six genes involved in the MVA pathway were analyzed at the transcriptional level, including *ACAT* encoding acetyl-CoA acetyltransferase, *HMGS* encoding HMG-CoA synthase, *HMGR* encoding HMG-CoA reductase, *MK* encoding mevalonate kinase, *PMK* encoding phosphomevalonate kinase, and *MPDC* encoding diphosphomevalonate kinase (Figure 2; Table 1) (Vranova et al., 2013). Heatmap analysis revealed that these genes exhibited different expression levels in the MVA pathway. However, only the genes *ACAT*, *HMGR*, *MK*, and *PMK* were decreased at 24 and 48 h under treatment with 50 µM mevinolin (Figure 3G), which correlated with the carotenoid and sterol contents (Figures 3D,F), suggesting their function in terpenoid accumulation. The transcription level of the other genes, *HMGS* and *MPDC,* was upregulated (Figure 3G). Mevinolin-stimulated *HMGS*, probably induced by the substrate HMG-CoA, may accumulate in the presence of the inhibitor. Furthermore, mevinolin-stimulated *MPDC*, probably induced by the product IPP, might not be detected.

To explore the effect of the MVA pathway inhibition on the synthesis and accumulation of lipids, the fatty acids of *Schizochytrium* were analyzed. TFA, polyunsaturated fatty acid (PUFA), and saturated fatty acid (SFA) of *Schizochytrium* increased by 15.24%, 27.44%, and 7.68%, respectively, with treatment with 50 µM mevinolin compared with the control (0 µM) (Figure 3H). This result indicates that inhibiting the terpenoid synthesis pathway induces lipid accumulation. HMGR inhibition in human cells also caused PUFA accumulation and activation of the genes involved in fatty acid production (Plée-Gautier et al., 2012). However, a higher concentration (100 µM) of mevinolin had a greater effect on sterol synthesis than lower concentrations (0–50 µM), especially the two sterols (*i.e.*, cholesterol and stigmasterol) that are essential components of the cell membrane. The huge changes in relative content and composition of sterols in mevinolin treated *Schizochytrium* cells might lead to the decreased stability and function of the cell membrane. In *Gemmata obscuriglobus,* the cell growth was repressed due to the inhibition of sterol synthesis, but again rescued with supplementation with exogenous lanosterol (Rivas-Marin et al., 2019). Thus, a higher concentration of mevinolin (100 µM) not only inhibited carotenoid and sterol synthesis but also impaired cell growth and fatty acid synthesis (Figures 3B,D,F,H).

These data suggest that owing to the lack of plastids in *Schizochytrium*, it may have used the MVA pathway to provide IPP/DMAPP for sterols and carotenoid syntheses, like fungi and animals. *Schizochytrium* cannot possibly assemble the MEP pathway through lateral acquisitions from the cyanobacterial ancestor of plastids (Lange et al., 2000). However, we cannot completely exclude the existence of the MEP or MEP-like pathway in *Schizochytrium*.

### 3.4 Putative use of widespread alternative squalene epoxidase in *Schizochytrium*

The flavoprotein squalene epoxidase catalyzes the stereospecific oxidation of squalene to 2,3-epoxysqualene, which is considered a rate-limiting enzyme in sterol biosynthesis (Jandrositz et al., 1991; Dong et al., 2018). Herein, no *SQE* (EC 1.14.14.17) orthologs were identified in the *Schizochytrium* genome*.* Moreover, the conventional *SQE* gene was not detected in the genomes of other algae species, including the *H. fermentalgiana* strain CCAP 4062/3 (Dellero et al., 2018), diatom *P. tricornutum* (Fabris et al., 2014), and brown alga *Aureococcus anophagefferens* (Desmond and Gribaldo, 2009). Apart from the conserved *SQE*, a widespread *AltSQE* (EC 1.14.19.-) was discovered, which belonged to the fatty acid hydroxylase superfamily (Pollier et al., 2019). The non-conserved *AltSQE* reportedly existed in numerous eukaryotic lineages; however, it was mutually exclusive with the conserved *SQE* and patchily distributed in monophyletic branches (Pollier et al., 2019). Subsequently, the amino acid sequence of *AltSQE* from *P. tricornutum* was used as a query to identify *AltSQE* from the *Schizochytrium* genome. One candidate *AltSQE* (Gene ID KN805388.1_orf00283) was identified in the *Schizochytrium* genome with an E-Value of 3E-49.

**FIGURE 4:**
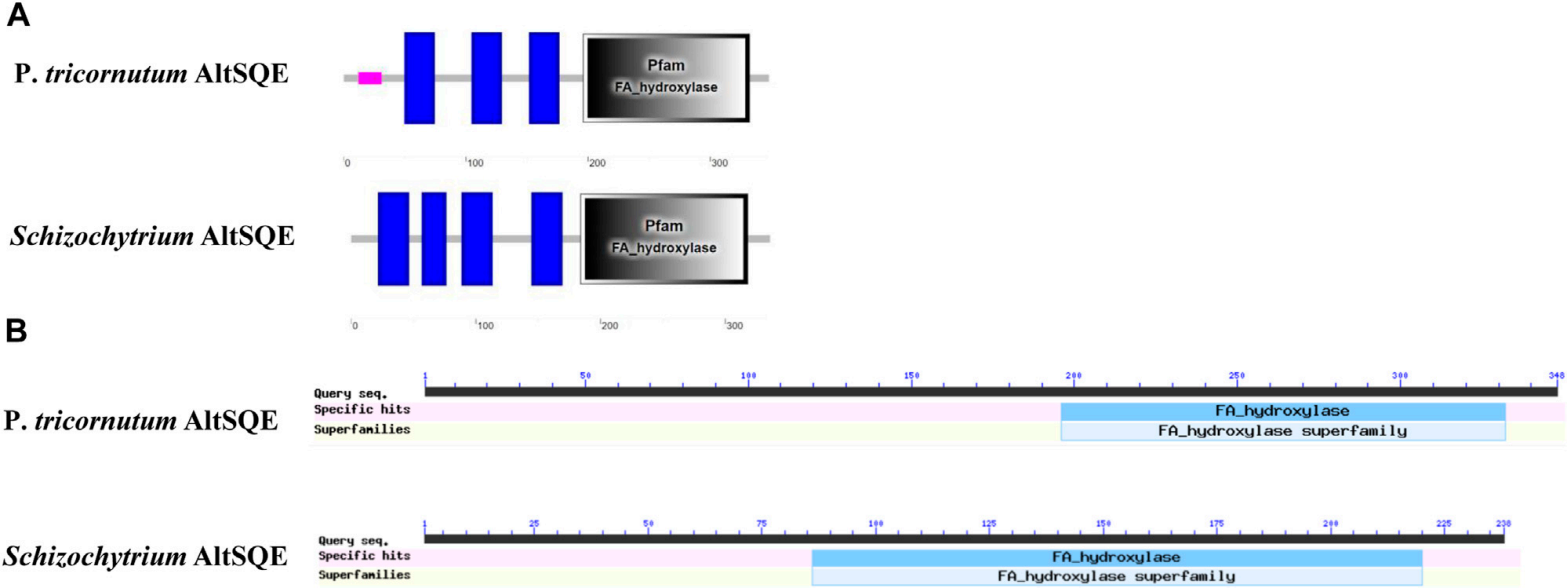
AltSQE protein domain analysis. **(A)**, protein domain analysis using SMART. **(B)**, protein domain analysis using The Conserved Domain Database. P. *tricornutum*: *Phaeodactylum tricornutum*. The protein domain analysis revealed that AltSQE from *Schizochytrium* belongs to the fatty acid hydroxylase superfamily, the same as AltSQE from *P. tricornutum.*

**FIGURE 5:**
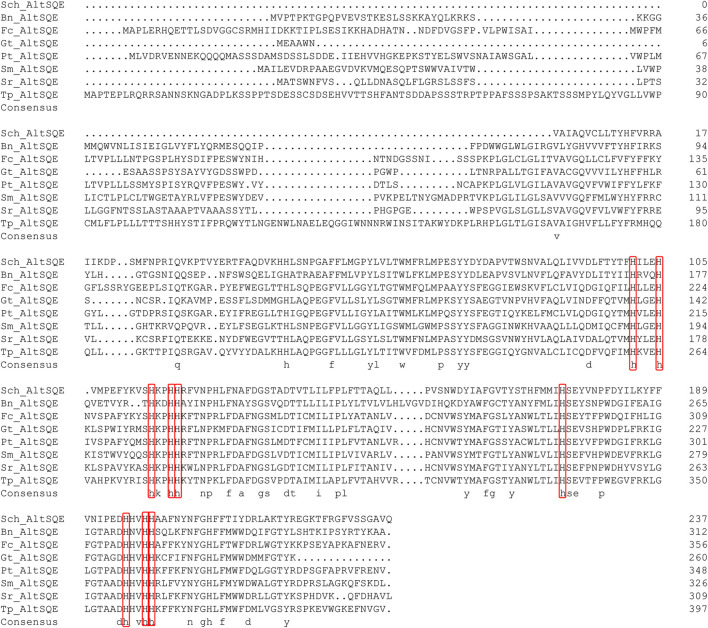
Sequence alignment of *Schizochytrium* AltSQE with AltSQE sequence from other species. DNAMAN is used for the alignment analysis. The conserved histidine residues are indicated in a red box. Abbreviations: Sch, *Schizochytrium*; Bn, *Bigelowiella natans*; Fc, *Fragilariopsis cylindrus*; Gt, *Guillardia theta*; Pt, *Phaeodactylum tricornutum*; Sm, *Symbiodinium minutum*; Sr, *Salpingoeca rosetta*; Tp, *Thalassiosira pseudonana*.

The protein domain analysis using SMART and the Conserved Domain Database revealed that the *Schizochytrium AltSQE* also belonged to the fatty acid hydroxylase superfamily following *P. tricornutum* (Figure 4). A common feature of the non-conserved *AltSQE* proteins belonging to the fatty acid hydroxylase superfamily is the nine conserved histidine residues, which coordinate a bimetal center at the active site of the enzyme (Bai et al., 2015; Zhu et al., 2015). Sequence alignment demonstrated that these residues were also conserved in *AltSQE* in *Schizochytrium* (Figure 5). These results suggest that *Schizochytrium* putatively possesses a non-conventional *AltSQE*, which catalyzes the stereospecific oxidation of squalene to 2,3-epoxysqualene in the sterol biosynthesis pathway.

**FIGURE 6:**
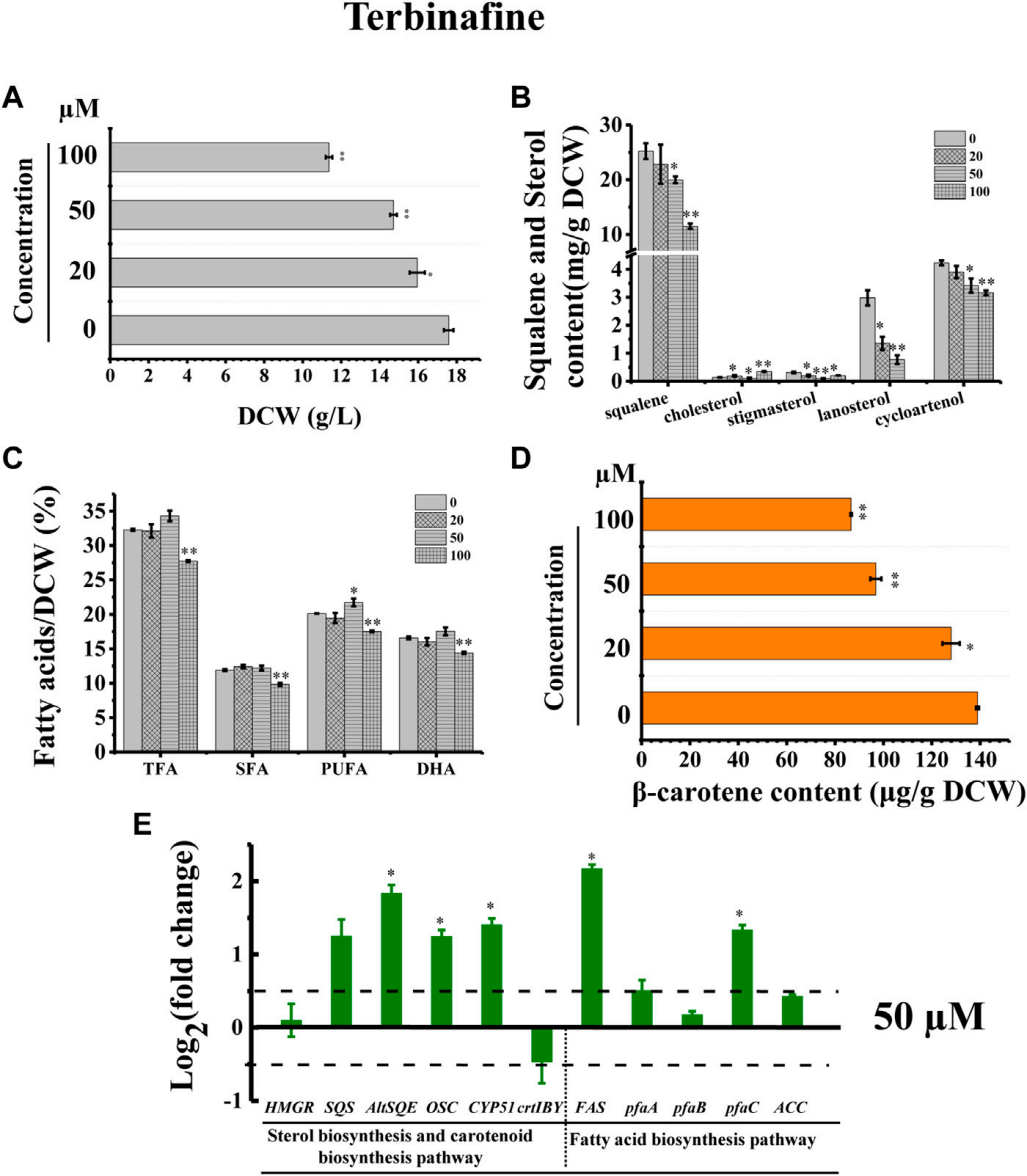
Effects of terbinafine on growth, sterols, lipids, and carotenoid in *Schizochytrium* sp.31. **(A)** DCW, **(B)** squalene and sterols, **(C)** fatty acids, and **(D)** β-carotene as affected by different concentrations (micromolar) of terbinafine. **(E)** Expression levels of selected genes involved in sterol, carotenoid, and fatty acid biosynthesis pathways with and without 50 µM terbinafine. The data are expressed as log2 (fold change) values of transcripts relative to the control (48 h), determined by qRT-PCR. Data represent means ± SD (n = 3). Asterisks (*) indicate *p* values <0.05. Asterisks (**) indicate *p* values <0.01. *HMGR*, hydroxy-methyl-glutaryl-CoA reductase; *SQS*, squalene synthase; *AltSQE*, squalene epoxidase; *OSC*, 2,3-oxidosqualene cyclase; *CYP51G1*, sterol 14-demethylase; *FAS*, fatty acid synthase; *pfaA*, polyunsaturated fatty acid synthase A; *pfaB*, polyunsaturated fatty acid synthase B; *pfaC*, polyunsaturated fatty acid synthase C; ACC, acetyl-CoA carboxylase.

In addition, we adopted a chemical biology approach to confirm the involvement of *AltSQE* in the *Schizochytrium* sterol pathway and measured the accumulation of the sterol pathway intermediates in *Schizochytrium* cultures treated with different concentrations of terbinafine, a specific inhibitor of squalene epoxidase (Wentzinger et al., 2002; Pollier et al., 2019) (Figure 2). Herein, three concentrations of terbinafine (20, 50, and 100 µM) were used to evaluate the responses of squalene and sterols in *Schizochytrium* (Figure 6). Terbinafine inhibited squalene and the two intermediate sterols (lanosterol and cycloartenol) synthesis in a concentration-dependent manner, and a mild effect on cholesterol and stigmasterol was observed with low dosages of terbinafine (20–50 µM) (Figure 6B). Transcriptional analysis of the key genes involved in sterol pathway revealed that the genes *SQS*, *AltSQE*, *OSC*, and *CYP51G1* were all upregulated under the treatment of 50 µM terbinafine (Figure 6E). However, a higher concentration (100 µM) of terbinafine significantly altered the synthesis of sterols. Cholesterol content doubled and lanosterol could not be detected following treatment with a higher concentration of terbinafine (100 µM) (Figure 6B), probably due to the lack of the precursor 2,3-oxidosqualene. However, a previous study reported that the squalene content was increased following terbinafine treatment at concentrations of 10 and 100 mg/L in *Aurantiochytrium mangrovei* FB3 (Fan et al., 2010). This different response of squalene accumulation in *Schizochytrium* and *A. mangrovei* might be due to different culture conditions and different adaptation models of the different strains following terbinafine treatment.

Furthermore, the cell growth of *Schizochytrium* was inhibited in a concentration-dependent manner, probably because of changes in sterols following treatment with terbinafine (Figure 6A). Conversely, terbinafine treatment induced fatty acids synthesis, leading to mild increases of 6.23%, 12.42%, and 7.99% for TFA, SFA, and PUFA of the DCW, respectively, at a concentration of 50 µM (Figure 6C), which was consistent with the findings of previous studies involving *N. oceanica* (Lu et al., 2014) and the diatom *P. tricornutum* (Fabris et al., 2014). And the transcriptional analysis of the five key genes involved in fatty acid synthesis revealed that the gene *FAS* and *pfaC* encoding polyunsaturated fatty acid synthase unit C were significantly upregulated under the treatment of 50 µM terbinafine, which might contribute to the increased fatty acid content (Figures 6C,E). A previous research showed that terbinafine treatment reduced sterol content and considerably elevated free fatty acid production in *N. oceanica* (Lu et al., 2014). In *P. tricornutum*, squalene 2,3-epoxide was confirmed as a sterol pathway intermediate using the SQE-specific inhibitor terbinafine and OSC-specific inhibitor Ro 48-8071 (Fabris et al., 2014). The addition of terbinafine also led to a decrease of sterol synthesis and an increase in fatty acid content in *P. tricornutum* (Fabris et al., 2014). However, a higher concentration of terbinafine (100 µM) attenuated fatty acid synthesis, probably owing to the lower cell metabolism resulting from huge changes in content and composition of sterols following to the treatment of 100 µM terbinafine (Figures 6B,C).

Furthermore, we found that the SQE-inhibitor terbinafine impaired carotenoids accumulations of *Schizochytrium* sp. in a concentration dependent manner, leading to significant decreases of 7.85%, 30.27%, and 37.61% for β-carotene of the DCW at the concentration of 20, 50, and 100 μM, respectively (Figure 6D). Transcriptional analysis revealed that the key gene *crtIBY* (encoding a trifunctional protein phytoene synthase/phytoene desaturase/lycopene cyclase) involved in carotenoid biosynthesis pathway (Gao et al., 2017; Tang et al., 2022) was downregulated at 48 h under treatment with 50 µM terbinafine, which is correlated with the carotenoid content change (Figures 6D,E). Previous studies have reported differential effects of terbinafine on carotenoid synthesis in other microorganisms. For instance, the addition of terbinafine reduced the sterols content but elevated the carotenoid content by 40% in *N. oceanica* (Lu et al., 2014). When 0.7 mg/L terbinafine was added to the culture medium at 48 h of fermentation, the lycopene content increased 23% in *Blakeslea trispora* (Sun et al., 2007). The different response of the carotenoid synthesis in different microorganisms suggested that terbinafine might have different effects between different microbial species, and there might be a novel regulation mechanism between sterols biosynthesis and carotenoid biosynthesis in *Schizochytrium*, which needs further investigation. Thus, these findings confirmed the involvement of *AltSQE* in the *Schizochytrium* sterol pathway and supported the link between sterol biosynthesis and lipid accumulation (Figure 6).

### 3.5 *SchOSC* putatively encodes a cyloartenol synthase

**FIGURE 7:**
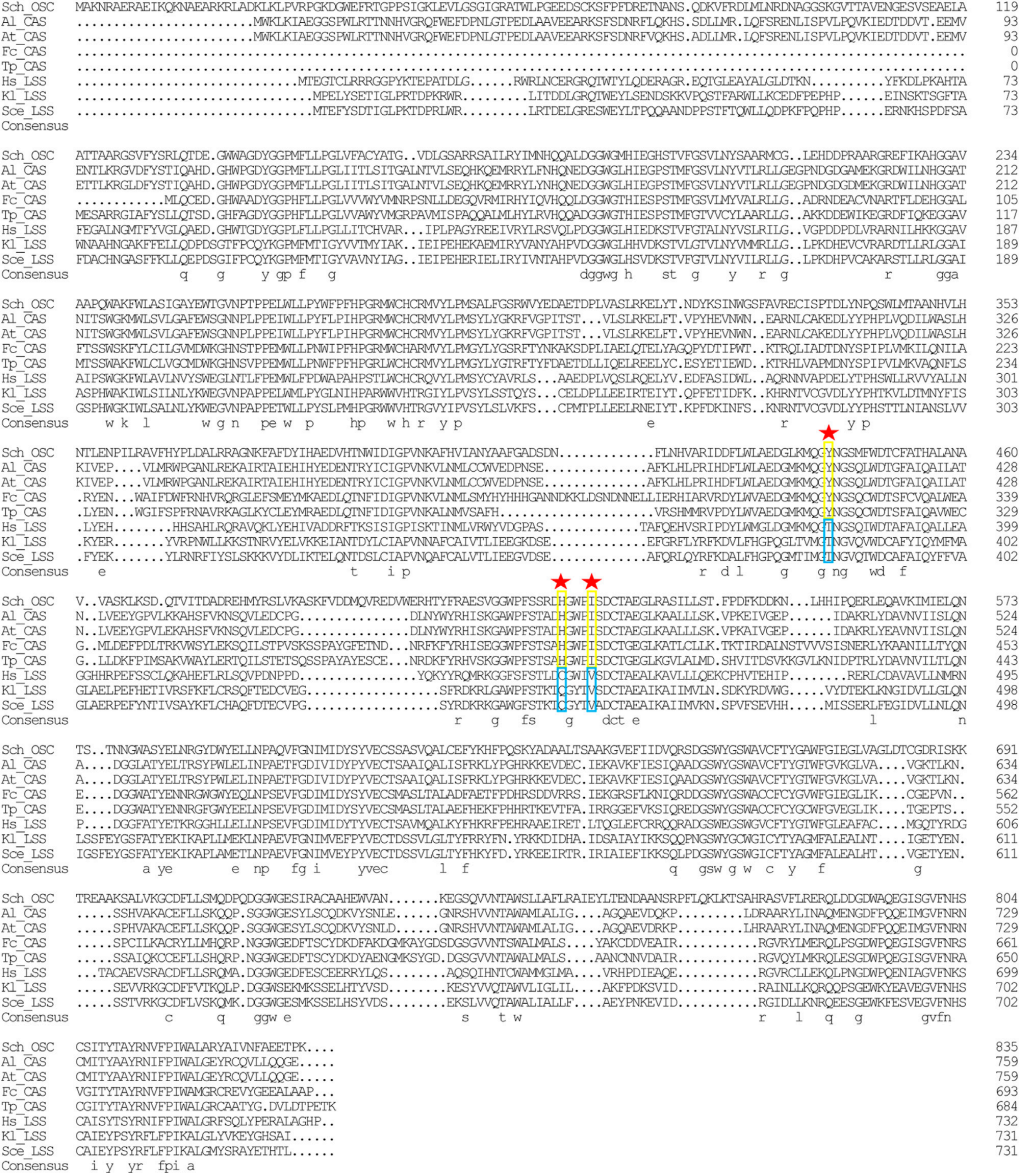
Sequence alignment of *Schizochytrium* OSC with OSC sequence from other species. DNAMAN is used for the alignment analysis. Arrowheads indicate positions in which conserved residues are specific for the formation of cycloartenol (yellow rectangles) or lanosterol (blue rectangles). Abbreviations: Sch, *Schizochytrium*; Al, *Arabidopsis lyrata*; At: *Arabidopsis thaliana*; Fc: *Fragilariopsis cylindrus*; Tp: *Thalassiosira pseudonana*; Hs: *Homo sapiens*; Kl: *Kluyveromyces lactis*; Sce: *Saccharomyces cerevisiae*.

In the sterol biosynthesis pathway, two distinct OSCs catalyze the cyclization reaction of 2,3-epoxysqualene. In animals and fungi, lanosterol synthase (*LAS*) catalyzed the cyclization of 2,3-epoxysqualene to lanosterol; in plants and green algae, cyloartenol synthase (*CAS*) catalyzed the cyclization of 2,3-epoxysqualene to cycloartenol (Fabris et al., 2014). Lanosterol and cycloartenol are important intermediate metabolites in the cholesterol and stigmasterol biosynthesis pathways, respectively (Fabris et al., 2014). Herein, stigmasterol, cholesterol, and its upstream intermediate metabolites (*i.e.*, cycloartenol and lanosterol) were detected in *Schizochytrium* sp. S31 (Figure 1A). However, only one copy of *SchOSC* (KN805388.1_orf0027) was detected and predicted *in silico* (Table 1). As OSC is a highly conserved enzyme, its specificity may be deduced from the amino acid residues at positions 381, 449, and 453 (numbering relative to the OSC of *H. sapiens*) that are required for producing cycloartenol (Y381, H449, I453) or lanosterol (T381, C/Q449, V453) (Figure 7) (Summons et al., 2006). The analysis of these active site residues in *SchOSC* suggested that *Schizochytrium* might use *SchOSC* to cyclize 2,3-epoxysqualene to cycloartenol acting as *CAS* (Figure 7), consistent with the previous postulation of *Thalassiosira pseudonana OSC* (Desmond and Gribaldo, 2009) and *P. tricornutum OSC* (Fabris et al., 2014). These results suggest that *SchOSC* putatively catalyzes the conversion of cycloartenol from 2,3-epoxysqualene. Whether *SchOSC* acts as a cycloartenol synthase or *LAS* requires further enzymological analysis.

### 3.6 Ketoconazole blocks the stigmasterol biosynthesis pathway of *Schizochytrium*

**FIGURE 8:**
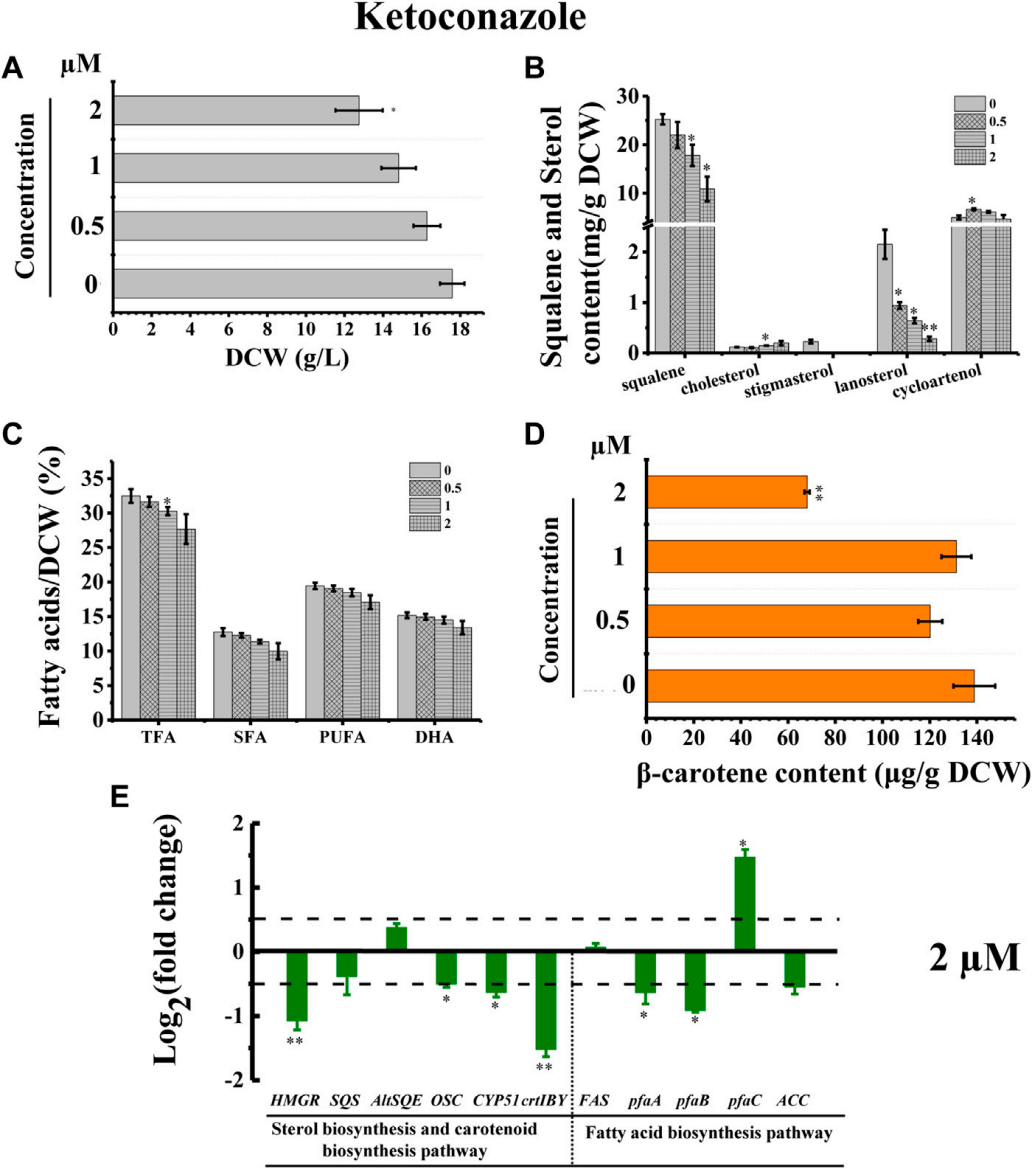
Effects of ketoconazole on growth, sterols, lipids, and carotenoid in *Schizochytrium* sp. S31. **(A)** DCW, **(B)** squalene and sterols, **(C)** fatty acids, and **(D)** β-carotene as affected by different concentrations (micromolar) of ketoconazole. **(E)** Expression levels of selected genes involved in sterol, carotenoid, and fatty acid biosynthesis pathways with and without 2 µM ketoconazole. The data are expressed as log_2_ (fold change) values of transcripts relative to the control (48 h), determined by qRT-PCR. Data represent means ± SD (n = 3). Asterisks (*) indicate *p* values <0.05. Asterisks (**) indicate *p* values <0.01.

 The inhibitor ketoconazole acts on sterol 14-α-demethylase (*CYP51G1*), a key regulatory enzyme for sterol biosynthesis (Warrilow et al., 2010). Ketoconazole affected the accumulation of both squalene and lanosterol in a concentration-dependent manner, decreasing squalene and lanosterol by 51.36% and 90.60%, respectively, at a concentration of 2 µM (Figure 8B). However, ketoconazole at a concentration of 2 µM (Figure 8B) induced cholesterol accumulation, increasing cholesterol by 80.76%. Additionally, ketoconazole completely blocked the stigmasterol biosynthesis pathway, as there was no stigmasterol detected following treatment with ketoconazole (Figure 8B). Transcriptional analysis revealed that the genes *HMGR*, *OSC*, and *CYP51G1* involved in sterol synthesis pathway were all significantly downregulated, which might contribute to the significantly decreased squalene, lanosterol, and stigmasterol synthesis (Figures 8B,E). As indicated in Figure 2, *CYP51G1* exists in both the cholesterol and stigmasterol pathways and acts as sterol 14-α-demethylase. However, the two reactions use two different substrates: lanosterol in the cholesterol biosynthesis pathway and obtusifoliol in the stigmasterol biosynthesis pathway. We speculated that ketoconazole might have a higher affinity for lanosterol than obtusifoliol, inducing the competitive inhibition of *CYP51G1*. However, the addition of ketoconazole inhibited cell growth (Figure 8A), possibly due to the severe effects of ketoconazole on sterol synthesis. The ketoconazole addition showed no significant effect on the fatty acid synthesis (Figure 8C). Transcriptional analysis of the key genes involved in fatty acid synthesis in *Schizochytrium* revealed differential regulations: *i*) the genes *pfaA* and *pfaB* were significantly downregulated; *ii*) the genes *FAS* and *ACC* showed no significant changes; *iii*) the gene *pfaC* was significantly upregulated at 48 h after 2 µM ketoconazole treatment compared with the control. We speculated that the diverse regulations of the key genes involved in fatty acid might result in a neutralizing effect, resulting in no significant change in fatty acid synthesis. Furthermore, the addition of ketoconazole at a concentration of 2 µM significantly reduced the β-carotene content by 51.01% compared to the control (Figure 8D). Transcriptional analysis revealed that both the two key genes (*HMGR* and *crtIBY*) were significantly downregulated under the treatment of 2 µM ketoconazole compared to the control, which correlated with the carotenoid contents (Figures 8D,E).

Stigmasterol is a phytosterol, which is the most essential component of plant cell membranes. Compared with cholesterol, stigmasterol has an additional double bond (Clayton et al., 1998). A previously study showed that phytosterol, including stigmasterol and campesterol, could order the two unsaturated fatty acyl chains of plant phospholipids more effectively due to the presence of the alkylated side chains, which can lead to the lower fluidity of membranes than cholesterol does (Leikin and Brenner, 1989). Thus, the loss of stigmasterol in ketoconazole-treated *Schizochytrium* cells might unbalance the fluidity and stability of cell membrane and decrease cell metabolism activity, contributing to decreased cell growth, and reduced fatty acid and carotenoid accumulation. According to a previously reported study, the sterol synthesis and the growth of the cultured *Leishmania mexicana* promastigotes could be inhibited by 50% under the treatment of ketoconazole at a concentration of 10^–2^ μM, which might be because of the interference with membrane permeability secondary to loss of desmethyl sterols and accumulation of 14α-methyl sterols (Berman et al., 1984). Thus, these findings confirmed the involvement of *CYP51G1* in the *Schizochytrium* sterol pathway.

## 4 Discussion

### 4.1 Construction of the *Schizochytrium* sterol biosynthesis pathway

The complete *Schizochytrium* sterol pathway was preliminarily elucidated based on the results of the inhibitor treatments. This pathway is suggested to be a hybrid of the algae and animal sterol biosynthesis pathways. Several elements of the predicted pathway were experimentally supported with data from chemical biological analysis and metabolite profiling of the wild-type. The study found that the *Schizochytrium* sterol biosynthesis pathway lacked conventional squalene epoxidase, and thus, probably involved a widespread alternative squalene epoxidase, which belonged to the fatty acid hydroxylase superfamily. The only copy of *SchOSC* putatively encodes cycloartenol synthase, catalyzing the conversion of 2,3-epoxysqualene into cycloartenol. Collectively, these data suggest that *Schizochytrium* utilizes a chimeric pathway to afford the main sterols cholesterol and stigmasterol. With the recent increases in the number of sequenced non-model and model organism genomes, and technological advances in genomics and metabolomics, the organization of sterol biochemistry and synthesis pathways have recently been re-evaluated (Massé et al., 2004; Kodner et al., 2008; Weete et al., 2010; Leblond and Lasiter, 2012; Miller et al., 2012; Nes et al., 2012; Fabris et al., 2014; Lu et al., 2014). The biochemistry of sterols and characterizations of sterol biosynthesis pathways of these organisms varied from the conventional model organisms, which enhanced our understanding of sterols. For example, bioinformatics analysis revealed that the sterol pathway of the oleaginous microalga *N. oceanica* also exhibited features of both plants and animals (Lu et al., 2014). Based on the phylogenomic analysis, enzyme inhibitor analysis, gene silencing, and heterologous gene analysis, the reconstructed sterol biosynthesis pathway of the diatom *P. tricornutum* displayed a mixture of features from plants and fungi (Fabris et al., 2014).

Based on our analysis, the following reaction scheme was postulated. First, squalene synthase (KN805399.1_orf01057, SQS) catalyzes the formation of squalene from farnesyl pyrophosphate. Then, a widespread alternative squalene epoxidase encoding by KN805388.1_orf00283 oxidizes squalene yielding 2,3-epoxysqualene. Although only a single 2,3-oxidosqualene cyclase (OSC) (KN805388.1_orf0027) was mined and was supposed to putatively encode cycloartenol synthase, catalyzing the formation of cycloartenol as the precursor of stigmasterol, such as in plants, *Schizochytrium* also generates lanosterol as a precursor of cholesterol, such as in animals. Subsequently, lanosterol and cycloartenol are catalyzed by the actions of a series of different enzymes to form cholesterol and stigmasterol, respectively.

Next, cycloartenol is methylated at C-24 by KN805382.1_orf01293, a sterol methyltransferase yielding 24-methylene-cycloartenol. Then, the methyl group at C-4 was removed by the subsequent actions of three enzymes, including 4,4-dimethylsterol C-4α-methyl-monooxygenase encoded by KN805429.1_orf00117, 3β-hydroxysteroid-4α-carboxylate 3-dehydrogenase encoded by KN805374.1_orf01215, and 3-keto-steroid reductase encoded by KN805408.1_orf00240, yielding cycloeucalenol. The resulting pentacyclic cyclopropyl sterol cycloeucalenol is then converted to tetracyclic sterol obtusifoliol via KN805375.1_orf02114 encoding cycloeucalenol cycloisomerase. Subsequently, the methyl groups at C-14 of obtusifoliol were removed by KN805454.1_orf00029, a sterol 14-demethylase, which shows significant similarity with plant orthologs. The C14-unsaturated bond of the resulting Δ-8-sterol is reduced by KN805390.1_orf00068 encoding Δ-(14)-Sterol reductase yielding Δ-8-sterol 4α-methylfecosterol, which is then converted to the corresponding Δ-7-isomer 24-methylenelophenol by KN805386.1_orf01801, a cholestenol Δ-isomerase. In addition, 24-methylenelophenol is methylated by KN805382.1_orf01293, and simultaneously, the other C-4 methyl is removed by the same three enzymes, including 4,4-dimethylsterol C-4α-methyl-monooxygenase, 3β-hydroxysteroid-4α-carboxylate 3-dehydrogenase, and 3-keto-steroid reductase, yielding Δ-7-avenasterol. Next, the resulting sterol was converted to stigmasterol through the subsequent redox reactions of four enzymes. The first was the Δ-7-sterol 5-desaturase encoded by KN805384.1_orf00665, similar to *H. sapiens* orthologs. The resulting 5-dehydroavenasterol was reduced via two sequential, enzymes 7-dehydrocholesterol reductase encoded by KN805434.1_orf00456 and Δ-24-Sterol reductase encoded by KN805380.1_orf01851, yielding sitosterol. Finally, C-22 sterol desaturase encoded by KN805393.1_orf00257 catalyzes sitosterol to form stigmasterol via the formation of the C-22 double bond.

In the proposed cholesterol biosynthesis pathway, the methyl groups at C-14 of lanosterol were first removed by KN805454.1_orf00029, a sterol 14-demethylase, yielding 4,4-dimethyl-5alpha-cholesta-8, 14,24-trien-3beta-ol. The C-14 unsaturated bond of the resulting sterol was subsequently reduced by Δ-(14)-sterol reductase encoded by KN805390.1_orf00068 to form 14-demethyllanosterol. In the following reaction, one of the C-4 methyl groups of 14-demethyllanosterol was also removed by the same sequential actions of three enzymes, including 4,4-dimethylsterol C-4α-methyl-monooxygenase encoded by KN805429.1_orf00117, sterol-4-α-carboxylate 3-dehydrogenase encoded by KN805374.1_orf01215, and 3-keto-steroid reductase encoded by KN805408.1_orf00240, yielding 4α-methylzymosterol. Subsequently, the same three enzymes catalyzed the resulting 4α-methylzymosterol to remove the other methyl group and convert it to zymosterol. Two different pathways converted zymosterol to cholesterol. Zymosterol was first converted to desmosterol via the sequential actions of three enzymes, including cholestenol Δ-isomerase encoded by KN805386.1_orf01801, Δ-7-sterol 5-desaturase encoded by KN805384.1_orf00665, and 7-dehydrocholesterol reductase encoded by KN805434.1_orf00456; then, desmosterol was converted to cholesterol by KN805380.1_orf01851 encoding Δ-24-sterol reductase. The other pathway involves the conversion of zymosterol into zymostenol by reducing the C-24 double bond via Δ-24-sterol reductase. Subsequently, zymostenol was converted to lathosterol by being isomerized by cholestenol Δ-isomerase encoded by KN805386.1_orf01801. Finally, lathosterol was converted to cholesterol through two redox reactions that involved 7-dehydrocholesterol reductase encoded by KN805434.1_orf00456 and Δ-24-sterol reductase encoded by KN805380.1_orf01851.

The elucidation of the complex *Schizochytrium* sterol pathway using the genome databases of *Schizochytrium* emphasized the significance and reliability of this approach for investigating *Schizochytrium* metabolism. The *Schizochytrium* sterol biosynthesis pathway possessed a chimeric feature of the algae/animal route and introduced possible novel enzyme substitutes for a highly conserved biochemical event. This highlighted the strong plasticity of *Schizochytrium* and introduced the possibility that the evolution of sterol biosynthesis in the marine environment might be different, thereby warranting a comprehensive reconsideration of the sterol synthetic pathway. *In vivo* or *in vitro* enzymatic analyses are required to uncover the alternative *LAS* and confirm the role of some of the sterol enzymes hypothesized in this study.

### 4.2 Active involvement of sterol metabolism in *Schizochytrium* growth

Together with glycerolipids and sphingolipids, sterols are crucial plasma membrane components for maintaining membranes in a microfluid state (Benveniste, 2004; Dufourc, 2008). In the oleaginous microorganism *Schizochytrium* sp. S31, the sterol biosynthetic pathway primarily produces cholesterol, stigmasterol, lanosterol, and cycloartenol. The profile of the sterols exhibited dynamic patterns during *Schizochytrium* cell proliferation. Sterols, particularly cholesterol and stigmasterol, are abundantly synthesized during the stationary phase of *Schizochytrium* cell proliferation (Figure 1C). The changes of the composition and relative content of these sterols in the cell membrane could affect the stability and fluidity of the cell membrane, which in turn affect the transmission of intracellular signals and the normal progress of cell life activities (Dufourc, 2008; Schaller, 2010). In *N. oceanica*, both the chemical blockages of 1-deoxy-D-xylulose 5-phosphate synthase using clomazone and SQE using terbinafine led to deformed cell morphology, which might due to the decrease of sterols under the treatment of sterol inhibitors (Lu et al., 2014). And, the chemical blockage of CPI1, CYP51G1, SQS, or SMT also led to the inhibition of cell proliferation (Lu et al., 2014). In *Arabidopsis*, the defect of *CYP51A2* changed the sterol content and sterol composition of the cell membrane, triggering the generation of reactive oxygen species and ethylene and eventually inducing seedling senescence (Kim et al., 2009). Thus, impairment in the growth of *Schizochytrium* cells whose HMGR, SQE, and CYP51G1 were blocked by specific chemical inhibitors may result from membrane structural defects due to a deficiency in specific sterols.

Based on all these results about *Schizochytrium* sterols, we can see that with the addition of sterol inhibitors, the intermediates (lanosterol and cycloartenol) were reduced, but cholesterol was significantly increased under all inhibition-induced conditions, especially at high concentrations of inhibitors (100 µM mevinolin, 100 µM terbinafine and 2 µM ketoconazole) (Figure 3D, Figure 6B, Figure 8B). In addition, the reduced accumulation of lanosterol and cycloartenol might largely due to the significant decreased supply of precursor squalene. The changes in cholesterol were a little unexpected, although the accumulation of their precursors was decreased. In higher plants, the membranes are affected by virous environmental condition and sterols play an important role in plant response to stress, particularly abiotic stress, such as ultraviolet (UV) radiation (Gil et al., 2012), low temperatures (Palta et al., 1993), and drought (Kumar et al., 2015). For instance, the levels of sitosterol and stigmasterol in young *Vitis vinifera* leaves were elevated following the low intensity UV-B (280–315 nm) treatment (16 h at 8.25 μW cm^− 2^) (Gil et al., 2012). In particular, the level of stigmasterol was increased 3.2-fold and 2.3-fold (exclusively in young leaves) under the low and high intensity UV-B radiation treatments, respectively, suggesting that UV-B radiation induced the synthesis of stigmasterol (Gil et al., 2012). The accumulation of β-sitosterol (the major phytosterol) in N22 (drought tolerant) rice seedlings was elevated proportionately with the severity of drought stress, indicating the role of phytosterol in providing tolerance to stress (Kumar et al., 2015). A previous study showed that suppression of *SMT1* encoding sterol-C24-methyltransferase 1 could induce the accumulation of cholesterol, which led to dwarfism and improved drought tolerance in herbaceous plants (Chen et al., 2018). Thus, the chemical inhibitors used in this study are not only inhibitors of enzymes involved in sterol synthesis pathway, but also might act as stress for *Schizochytrium* as those inhibitors greatly altered the content and composition of sterols, fatty acids, and carotenoids in *Schizochytrium* cells. We therefore speculate that cholesterol may play an important role in response to the stress induced by sterol inhibitors in *Schizochytrium*.

The inhibition of HMGR, SQE, and CYP51G1 is associated with a defect in carotenoid synthesis in *Schizochytrium*. Both the sterol biosynthesis and carotenoid biosynthesis pathways use the MVA pathway to provide the common precursor IPP (Kuzuyama and Seto, 2012). Thus, the inhibition of HMGR activity with the addition of mevinolin led to not only the decreased sterols, but also the decreased β-carotene accumulation, which was also supported by the downregulated gene expression of *HMGR* (Figures 3D,F,G). Especially when CYP51G1 was inhibited by the inhibitor ketoconazole at a concentration of 2 μM, both of the genes *HMGR* and *crtIBY* were significantly downregulated, which might contribute to the significant defect of β-carotene synthesis (Figures 8D,E). Although the chemical blockage of SQE using terbinafine also led to reduced β-carotene accumulation, the transcription levels of *HMGR* and *crtIBY* were not significantly regulated (Figures 6D,E). However, previous studies have reported differential effects of terbinafine on carotenoid synthesis in other microorganisms (Sun et al., 2007; Lu et al., 2014). The addition of terbinafine could improve the carotenoid synthesis in *N. oceanica* (Lu et al., 2014) and in *B. trispora* (Sun et al., 2007). The different responses of the carotenoid synthesis to terbinafine in different microorganisms suggested that terbinafine might have different effects between different microbial species, and there might be a novel regulation mechanism between sterols biosynthesis and carotenoid biosynthesis in *Schizochytrium*. We therefore speculate that the inhibition of sterol synthesis might trigger reduced carotenoid synthesis and accumulation in *Schizochytrium*.

### 4.3 Feedback regulation of sterol biosynthesis and role of sterols in lipid homeostasis

Co-regulation of fatty acid accumulation and sterol biosynthesis is essential to keep the balance between membrane biosynthesis and turnover during normal cellular growth (Bennett et al., 1995). The synthesis and accumulation of cholesterol in mammals is highly regulated. Over-accumulation of cholesterol inhibits the synthesis of fatty acids and cholesterol, whereas low levels of cholesterol promote it (Bengoechea-Alonso and Ericsson, 2007). The co-regulation of sterol and fatty acid biosynthesis might also exist in *Schizochytrium*. Overexpression of ω-3 desaturase led to an elevated ratio of ω-3/ω-6 fatty acids, but a reduced accumulation of squalene and sterols in *Schizochytrium* sp. HX-308 (Ren et al., 2015). Overexpression of the acetyl-CoA acetyltransferase gene improved the terpenoid (sterols and carotenoid) biosynthesis, yet decreased the TFA by 71% compared to the control in *Schizochytrium* sp. HX-308 (Huang et al., 2021). In this work, we showed that chemical blockage of HMGR and SQE of *Schizochytrium* improved the synthesis of fatty acid with appropriate concentrations (20–50 µM) of inhibitors (mevinolin and terbinafine) (Figure 3H, Figure 6C). On the contrary, a higher concentration (100 µM) of mevinolin or terbinafine in the fermentation medium inhibited the synthesis and accumulation of fatty acid (Figure 3H, Figure 6C). And, the addition of ketoconazole, an inhibitor of CYP51G1, decreased the sterol synthesis, but showed no significant effect on the fatty acid accumulation (Figure 8C). The reason for this phenomenon might be that the huge changes in content and composition of sterols affected by inhibitors led to the lower fluidity and stability of cell membrane and decreased cell metabolism activity.

An appropriate composition of sterols in the cell membrane is very important for optimal enzymatic activity, ion and metabolite transport or channeling, protein–protein and protein–lipid interactions, signal transduction and to deal with fluctuating environmental conditions (Schaller, 2003). In general, significant disruptions to the relative content of sterols in cell membranes can have detrimental effects on the cell’s health and survival (Schaller, 2003; Rivas-Marin et al., 2019). Growth of *G. obscuriglobus* was repressed by treatment with terbinafine or zaragozic acid, an inhibitor of squalene synthase, which was due to the inhibition of sterol synthesis, but again rescued with supplementation with exogenous lanosterol (Rivas-Marin et al., 2019). However, the *G. obscuriglobus* mutant cells with the knockout of the genes *SQE* and *OSC* were unable to sustain normal unlimited growth (Rivas-Marin et al., 2019). In plant *Arabidopsis*, vegetative development, such as stem elongation, is only slightly impacted when the ratio of campesterol to sitosterol is 30 times greater than in the wild-type plant, while sexual reproduction is significantly impacted when the ratio of campesterol to sitosterol changes by a factor of 10 (Schaller, 2003).

In this study, the lanosterol could not be detected from mevinolin or terbinafine treated *Schizochytrium* cells at a concentration of 100 µM (Figure 3D, Figure 6B), suggesting that lanosterol was in short supply in *Schizochytrium*. Lanosterol is an important sterol, serving as a precursor to other sterols such as cholesterol in mammals and ergosterol in yeast and fungi (Miao et al., 2002; Rivas-Marin et al., 2019). Ketoconazole also blocked the stigmasterol biosynthesis pathway of *Schizochytrium* (Figure 8B). Stigmasterol plays an important role in plant growth and development. The intracellular balance of sitosterol and stigmasterol is of great physiological importance for plant growth and development, as well as for the stress response (Rogowska and Szakiel, 2020). Fluctuations in the sterol composition, such as changes in the ratio of sitosterol to stigmasterol, may be essential for certain processes relating to plant growth and development (Rogowska and Szakiel, 2020). We, therefore, speculate that inhibition of sterol synthesis could improve fatty acid synthesis in *Schizochytrium,* but only if the changes in sterol content and composition of *Schizochytrium* cell membrane do not affect the homeostasis of cell membranes, otherwise it will have the opposite effect.

## 5 Conclusion

In summary, sterols play important roles in *Schizochytrium* growth, carotenoid synthesis, and fatty acid synthesis. The sterol biosynthesis pathway of *Schizochytrium* was elucidated using genome analysis and a chemical biology approach, which revealed a chimeric organization of the sterol pathway. Sterol and carotenoid metabolisms are possibly co-regulated, such that inhibition of sterols led to decreased carotenoid synthesis through down-regulating the gene *HMGR* and *crtIBY* in *Schizochytrium*. In addition, the inhibition of sterol synthesis could promote the accumulation of fatty acid in *Schizochytrium.* Understanding these regulatory mechanisms may lead to rational strategies for improved growth characteristics for *Schizochytrium* under challenging environmental conditions and for the development of improved feedstocks for DHA or other high-value chemicals.

## Data Availability

The datasets presented in this study can be found in online repositories. The names of the repository/repositories and accession number(s) can be found in the article/Supplementary Material.

## Supplementary material

SUPPLEMENTARY FIGURE S1 EI-MS spectra of the main peaks of the chromatograms shown in Figure 1A and that of the corresponding authentic standards. The EI-MS of peak 5 possibly corresponds to TMS-gramisterol, for which no standard was available.
