# Supplementary figures and images for: Elucidation of sterol biosynthesis pathway and its co-regulation with fatty acid biosynthesis in the oleaginous marine protist Schizochytrium sp
Source: Front Bioeng Biotechnol. 2023 Apr 27;11:1188461. doi: 10.3389/fbioe.2023.1188461 (PMC10174431; doi:10.3389/fbioe.2023.1188461)

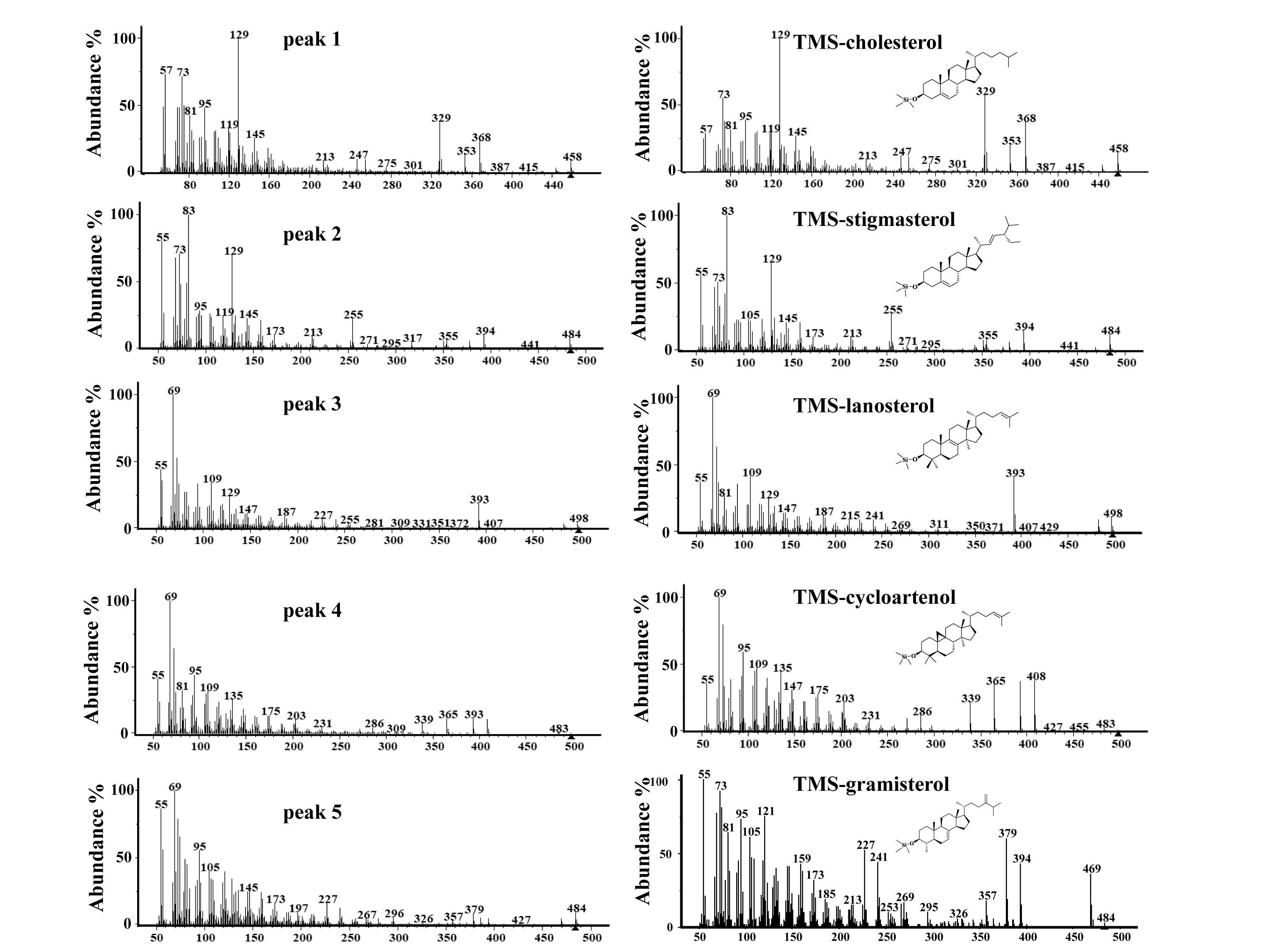

Supplement: Supplementary file 2 [file Image1.TIF]
